# Evolution from Covalent to Self-Assembled PAMAM-Based Dendrimers as Nanovectors for siRNA Delivery in Cancer by Coupled in Silico-Experimental Studies. Part II: Self-Assembled siRNA Nanocarriers

**DOI:** 10.3390/pharmaceutics11070324

**Published:** 2019-07-10

**Authors:** Erik Laurini, Domenico Marson, Suzana Aulic, Maurizio Fermeglia, Sabrina Pricl

**Affiliations:** Molecular Biology and Nanotechnology Laboratory (MolBNL@UniTS), Department of Engineering and Architecture, University of Trieste, 34127 Trieste, Italy

**Keywords:** RNAi therapeutics, siRNA delivery, amphiphilic dendrons, PAMAM dendrimers, self-assembling, nanovectors, gene silencing

## Abstract

In part I of this review, the authors showed how poly(amidoamine) (PAMAM)-based dendrimers can be considered as promising delivering platforms for siRNA therapeutics. This is by virtue of their precise and unique multivalent molecular architecture, characterized by uniform branching units and a plethora of surface groups amenable to effective siRNA binding and delivery to e.g., cancer cells. However, the successful clinical translation of dendrimer-based nanovectors requires considerable amounts of good manufacturing practice (GMP) compounds in order to conform to the guidelines recommended by the relevant authorizing agencies. Large-scale GMP-standard high-generation dendrimer production is technically very challenging. Therefore, in this second part of the review, the authors present the development of PAMAM-based amphiphilic dendrons, that are able to auto-organize themselves into nanosized micelles which ultimately outperform their covalent dendrimer counterparts in in vitro and in vivo gene silencing.

## 1. Self-Assembling PAMAM-Based Amphiphilic Dendrons: A New Paradigm for siRNA Delivery

In its broadest sense, self-assembly describes the natural tendency of physical systems to exchange energy with their surroundings and assume patterns or structures of low free energy. Random thermal motions bring constituent particles together in various configurations, and only those with significantly favorable interaction energy forming, tend to persist, and eventually become predominant. The information on the shape and size of the ultimate self-assembled entity is embodied in the structures of the individual components. A system slowly approaching equilibrium assumes a simple repetitive structure, while a dynamic system may generate structures of great complexity. For example, molecules in a cooling glass of water self-assemble as simple ice crystals, while the same molecules in a turbulent cloud with temperature and humidity gradients self-assemble as complex snowflakes of enormous variety. In relation to chemistry, self-assembly constitutes the quintessence of nanotechnology-based techniques leading to the design of novel materials [1,2,3,4]. It relies on the cumulative effects of multiple non-covalent interactions to assemble molecular building blocks into supramolecular entities in a reversible, controllable, and specific way, yet with relatively little synthetic effort. In particular is the ability of self-assembled structures to behave as more than the sum of their individual parts, and exhibit completely new properties [5].

With these concepts in mind, the authors envisaged the idea of creating small amphiphilic poly(amidoamine) (PAMAM)-based dendrons which, upon auto-organization into nanosized micelles, could mimic the covalent, high-generation dendrimer counterparts in size, shape and function, in particular for in vitro and in vivo siRNA delivery [6,7,8], as set out below.

## 2. siRNA Delivery by Single-Tail Self-Assembling Amphiphilic Dendrons

### 2.1. Design, Optimization and Chemico-Physical Characterization of Single-Tail Self-Assembling Amphiphilic Dendrons

**Scheme 1:**
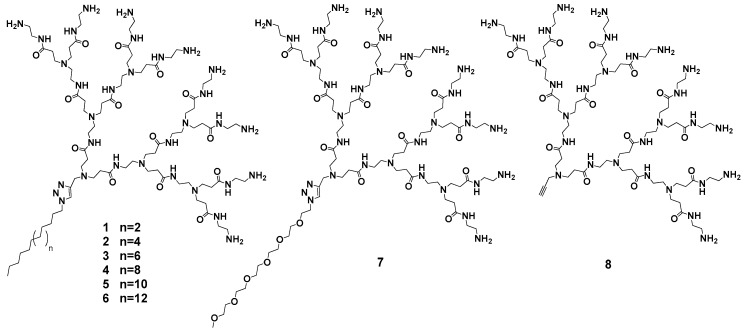
The chemical structure of the PAMAM-based self-assembling amphiphilic dendrons bearing a single alkyl chain of variable length (**1**–**6**). The two non-amphiphilic dendrons characterized by a hydrophilic pentaethylene glycol (PEG) chain (**7**) and by the presence of the sole PAMAM head (**8**) used as negative reference compounds are also shown (see text for details). Adapted from [7] with the permission of John Wiley and Sons, 2016.

Our work in this field started with the design, optimization and synthesis of a series of amphiphilic dendrons (**1**–**6**) characterized by a single hydrophobic alkyl chain of variable length and a hydrophilic PAMAM head with 8 primary amine terminal groups [7], as shown in Scheme 1. In addition, two non-amphiphilic dendrons characterized by a hydrophilic pentaethylene glycol (PEG) chain (**7**) and by the presence of the sole PAMAM head **(8**) were also produced as negative reference compounds.

**Figure 1:**
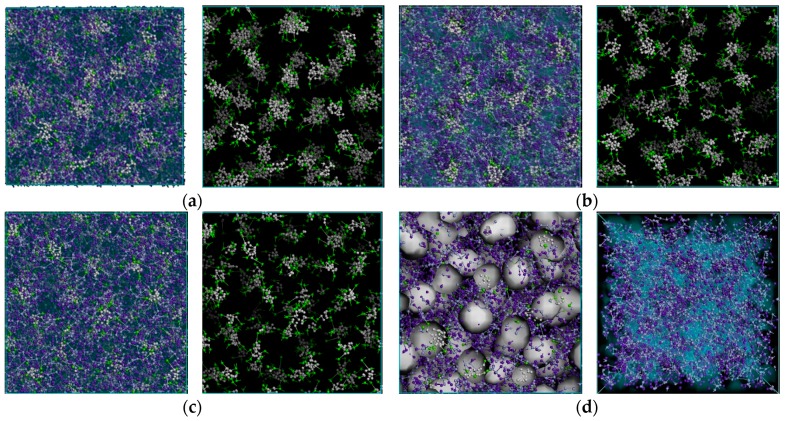
The computer simulation of the self-assembled micelles generated by the amphiphilic dendrons **4** (**a**), **3** (**b**), and **2** (**c**). In all panels, the hydrophilic PAMAM head is portrayed as purple and lilac beads, the hydrophobic tail is represented by light gray beads while the linker between the hydrophilic and the hydrophobic portion of the molecule is shown as green beads. Water and counterions are portrayed as a gray field for clarity. The right images in each panel show only the micellar cores. (**d**, **left**) Zoomed view of the micelles formed by dendron **4**. The micellar cores are highlighted by a light gray surface. (**d**, **right**) Computer simulation of the non-amphiphilic dendron **8** selected as negative control, showing the non-self-assembling characteristics of this system.

Mesoscale simulations (see Figure S1, Table S1, and text in Supporting Information for details) were initially employed to predict the self-assembly of all dendrons **1**–**8**. According to the in silico results, the amphiphilic dendrons **1**–**6** were able to auto-organize into spherical nanosized micelles (shown in Figure 1 for dendrons **4**, **3** and **2** as an example), with an average diameter D and aggregation number N_agg_ ranging from 5.6 nm and 5 for micelles. This was generated by dendron **1** to 8.1 nm and 13 for those originated by the self-assembling of dendron **6** (Table 1). Contextually, no stable nanostructure formation was observed for the negative controls **7** and **8**, as shown in the left panel of Figure 1d for dendron **8**.

The values of the critical micelle concentration (CMC) for the amphiphilic dendrons **1**–**6** (see Supporting Information for details) were further investigated from which a clear inverse relationship between the hydrophobic tail length and the CMC was obtained (Table 1). In other words, the amphiphilic dendrons bearing a longer hydrophobic component are able to self-assemble and pack more efficiently than those characterized by shorter alkyl tails. In addition, the associated values of the free energy of micellization (ΔG_m_) predicted by simulation (see Supporting Information for details) were all largely negative, supporting the spontaneous and thermodynamically favored micelle formation for all dendrons **1**–**6** (Table 1). As the hydrophilic portion was the same in all amphiphilic dendrons, the differential contribution to ΔG_m_ (and hence to CMC) is obviously related to the length of the hydrophobic chain.

**Figure 2:**
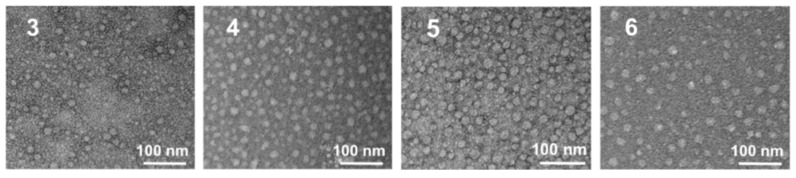
TEM images of spherical micelles formed by the amphiphilic dendrons **3**–**6**. Adapted from [7] with the permission of John Wiley and Sons, 2016.

Experimental confirmation of in silico predictions were first performed by dynamic light scattering (DLS) and transmission electron microscopy (TEM). Both these techniques confirmed that the amphiphilic dendrons predominantly formed nanosized spherical micelles, as shown in Figure 2 for dendrons **3**–**6**. In particular, data analysis confirmed that an increase of the hydrophobic tail length from C_14_ (**3**) to C_22_ (**6**) was paralleled by an increase of the micellar diameter D_exp_ from 6.6 nm to 7.8 nm, in full agreement with the computer-based predictions (Table 1). Quite interestingly, the overall dimensions of these micellar nano-objects are indeed very close to those characterizing the high-generation structurally flexible triethanolamine (TEA)-core covalent dendrimers discussed in detail in the companion paper [9] (e.g., the average molecular diameters for generation 4, 5 and 6 TEA-core dendrimers are equal to 5.2, 7.7 and 10.1 nm, respectively). Therefore, at least from the standpoint of structure and dimensions, the self-assembled nanoparticles obtained from the amphiphilic dendrons can be considered as supramolecular dendrimer mimics.

The predicted CMC values were also confirmed by experiments using pyrene as a hydrophobic fluorescent probe (Table 1). The progressive increase in alkyl chain length resulted in a drop of CMC_exp_ from 398 μM for dendron **1** to 8.24 μM for dendron **6**. The high values of the computational/experimental CMC for systems **1** and **2** are the likely reason why micelle formation by these two dendrons was not observed under the conditions adopted for DLS/TEM experiments. Further, in agreement with the simulation, no CMC value could be experimentally determined for **7** and **8**, confirming the non-self-assembling properties of these two negative-control systems.

### 2.2. In Silico/Experimental Interaction of Single Tail Self-Assembling Dendrons and siRNA

**Figure 3:**
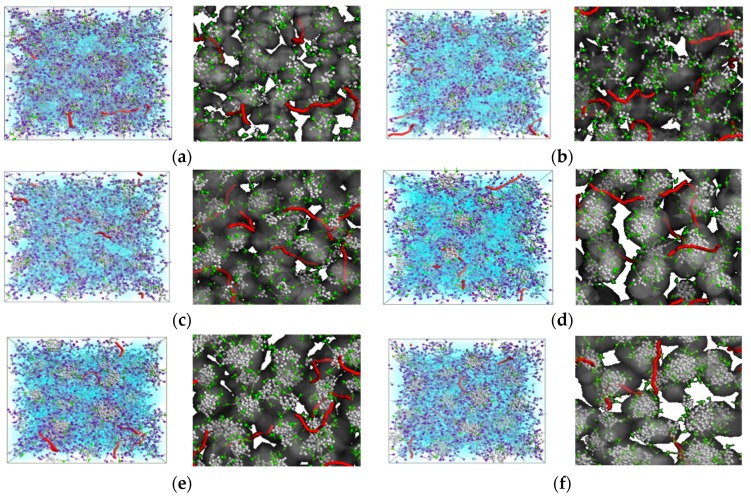

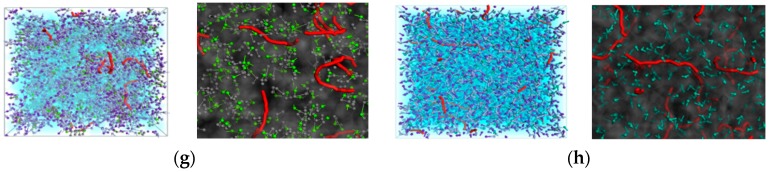
Morphologies of the systems formed at a dendrimer-to-siRNA charge ratio (N/P) = 10 by the siRNA molecules and the amphiphilic dendrons **1** (**a**), **2** (**b**), **3** (**c**), **4** (**d**), **5** (**e**), **6** (**f**), **7** (**g**) and **8** (**h**) as obtained from molecular simulations. Left panels are zoomed images of the right panels. Colors as in Figure 2, except for the siRNA molecules, shown as red sticks and the water and counterions, portrayed in the left panels as light blue field for clarity. Adapted from [7] with the permission of John Wiley and Sons, 2016.

Mesoscale simulations were again exploited for predicting the interactions between the amphiphilic dendrons **1**–**8** and siRNA molecules, as illustrated in Figure 3.

The computer images showed that, at the same dendrimer-to-siRNA charge ratio (N/P) of 10, the nucleic acid fragments are well encased within the micellar network formed by all self-assembling amphiphilic dendrimers **1**–**6** and so are efficiently protected from the surrounding environment. For the non-self-assembling systems **7** and **8**, the siRNA molecules are interspersed within an unorganized solution of single dendrons and water, and therefore in principle more susceptible to ultimate degradation by e.g., RNAses.

The in silico quantitative characterization of the siRNA/amphiphilic dendron interactions was next carried out by atomistic molecular dynamics (MD) simulations performed in the framework of the so-called molecular mechanics/Poisson-Boltzmann surface area (MM/PBSA) methodology [9,10,11,12,13,14,15] (the methodology is described in detail in the Supporting Information in the companion paper [9]). The values of free energy of binding (ΔG_bind_) between the different self-assembling dendrons **1**–**6** towards the siRNA sequence directed against the mRNA coding for the heat shock protein 27 (Hsp27)—a small molecular chaperone which is a vital regulator of cell survival and a major player in drug resistance—were initially calculated, as listed in the first column of Table 2. However, in order to perform a rigorous discussion about these aspects, the concept of effective free energy of binding (ΔG_bind_,_eff_) was introduced, as follows. From an extensive analysis of each dendron micelle/siRNA complex MD trajectory, the number N_eff_ of dendron branches (each bearing a positive charge) in permanent contact with the siRNA was determined, leading to the values listed in the second columns of Table 2. The precise identification of each dendrimer branch involved in the siRNA binding, and the corresponding individual contribution afforded to the overall binding energy, was then carried out by a per-residue free energy decomposition technique (described in the Supporting Information in the companion paper [9]). The cumulative results of this analysis are reported in the third column of Table 2, showing the effective contribution to the total free energy of binding (ΔG_bind,eff_). However, to be able to compare simulation data among themselves (and with experimental data, see below) the effective-charge-normalized values of ΔG_bind,eff_, i.e., ΔG_bind,eff_/N_eff_, were considered, as shown in the fifth column of Table 2.

From the values reported in Table 2, the effective interaction between the micelles formed by the different dendrons with siRNA (ΔG_bind,eff_) increases with increasing alkyl chain length, the last three dendrons definitely being the strongest siRNA binders in the order: **4** < **5** < **6**. Limiting the discussion to these three self-assembling systems, the micelles formed by dendron **4** were characterized by a total number of charges equal to +64, the 48% of which (N_eff_ = 31 are involved in productive siRNA binding. This translates into the corresponding ΔG_bind,eff_/N_eff_ value of −0.41 kcal/mol. In contrast, the other two efficient self-assembled micelles generated by dendrons **5** and **6** were not only both able to exploit 61% of their positive charges (49/80 and 62/102 for **5** and **6**, respectively) but they also did it more efficiently (ΔG_bind,eff_/N_eff_ = −0.59 kcal/mol for **5** and −0.56 kcal/mol for **6**, Table 1), ultimately characterizing the corresponding micelles as the best siRNA binders of the whole series.

**Figure A1:**
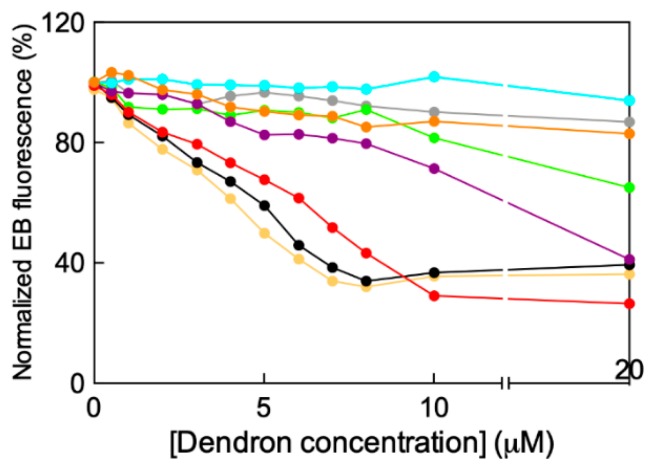
Experimental determination of siRNA binding to self-assembling dendrons **1** (gray), **2** (light green), **3** (purple), **4** (red), **5** (black), and **6** (yellow) by EB assay. Non-self-assembling dendrons **7** (cyan) and **8** (orange) were used as negative controls. Adapted from [7] with the permission of John Wiley and Sons, 2016.

In silico data were experimentally confirmed by fluorescent ethidium bromide (EB) displacement assays (see Figure A1 in Appendix A). According to these tests, the value of C_50_ (i.e., the dendron concentration at which the fluorescence of siRNA-intercalated EB is reduced by 50%), is an indication of siRNA binding efficiency: The lower the C_50_ value, the stronger the siRNA/micelle binding. As seen in Figure A1, the negative-control (i.e., non-self-assembling) dendrons **7** and **8** were not able to displace EB from the nucleic acid. In line, also for the self-assembling dendron **1**, bearing the shortest alkyl tail (C_14_), the C_50_ value was not determined, due to the high CMC value required for its self-assembly. On the other hand, for the dendron series **2**–**6**, progressively decreasing C_50_ values were obtained (Figure A1 and Table 2, last column), with dendron **6** being the most effective siRNA binder, in full agreement with computer-based predictions.

### 2.3. In Vitro siRNA Delivery Performance of Single-Tail Self-Assembling Dendrons

**Figure 4:**
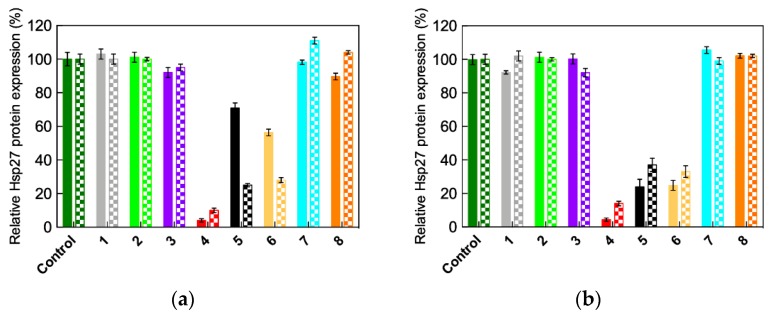

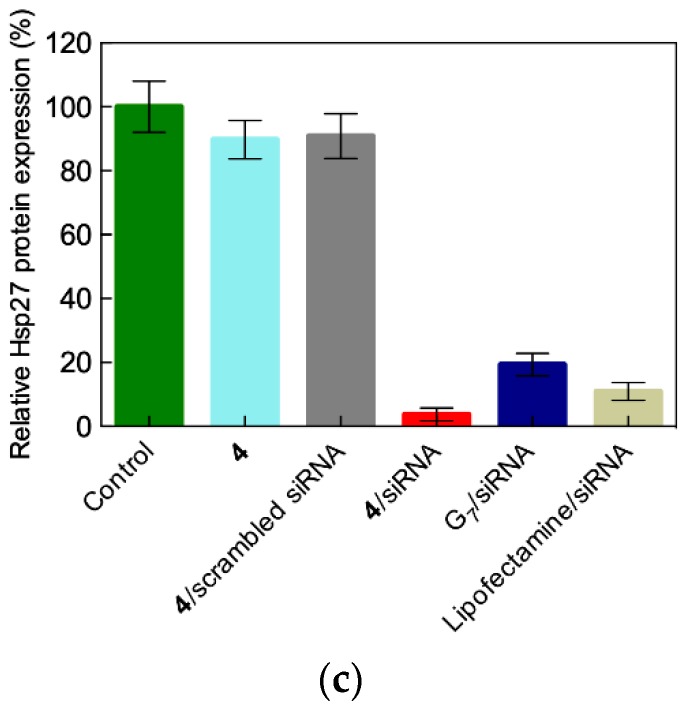
Self-assembled dendron-mediated siRNA delivery and gene silencing of Hsp27 (**a**) and TCTP proteins (**b**) in human prostate cancer PC-3 (solid bars) and C4-2 (patterned bars) cell lines (N/P = 10). Control: Untreated cells. The results obtained from non-self-assembling dendrons **1**, **7** and **8** are also shown for negative-control purposes. (**c**) Expression of Hsp27 in PC-3 cells treated with 50 nM siRNA delivered by self-assembled dendron **4** (N/P = 10), by Lipofectamine, and by the high-generation covalent triethanolamine (TEA)-core PAMAM-based dendrimer G_7_. Control: Untreated cells. Negative controls: Self-assembled dendron **4** alone and a scrambled (i.e., non-silencing) siRNA sequence. The expression levels of Hsp27 and TCTP proteins were quantified by western blots 72 h post treatment using vinculin as the control. Adapted from [7] with the permission of John Wiley and Sons, 2016.

Based on the results described above, dendrons **4**–**6** were selected for in vitro delivery of siRNA molecules targeting both the Hsp27 and the translationally controlled tumor protein (TCTP, a highly conserved protein present in all eukaryotic organisms that regulate cell survival in human tumors) in human castration-resistant prostate cancer (HCPC) PC-3 and C4-2 cell lines (Figure 4a,b). In these experiments, all other dendrons (**1**, **3**, **7** and **8**) were also tested for comparison and/or negative controls.

As shown in this Figure, the best siRNA delivery capacity was obtained with the nanovector based on the self-assembled form of dendron **4**, whose related gene silencing effect was even superior to that obtained both with the gold standard commercial vector Lipofectamine and with the high-generation (G_7_) covalent PAMAM-based dendrimer. This featured an extended triethanolamine (TEA) core developed by our group and discussed in detail in the companion paper [9]. As anticipated by in silico predictions, nanocarriers formed by dendrons bearing shorter aliphatic (**1**–**3**), hydrophilic (**7**) or no chains (**8**) fail to elicit meaningful gene silencing in all cases. Indeed, while **7** and **8** are intrinsically unable to self-assembly (negative controls), the former dendron set (**1**–**3**) likely cannot generate stable micelles and/or siRNA complexes at the experimental conditions employed for transfection, due to their high CMC and C_50_ values (Table 1 and Table 2). On the other hand, nanomicelles from dendrons with longer alkyl tails (**5** and **6**) were also able to achieve significant siRNA delivery and silenced the target Hsp27 and TCTP genes, yet with performance lower than that obtained with dendron **4**.

**Figure 5:**
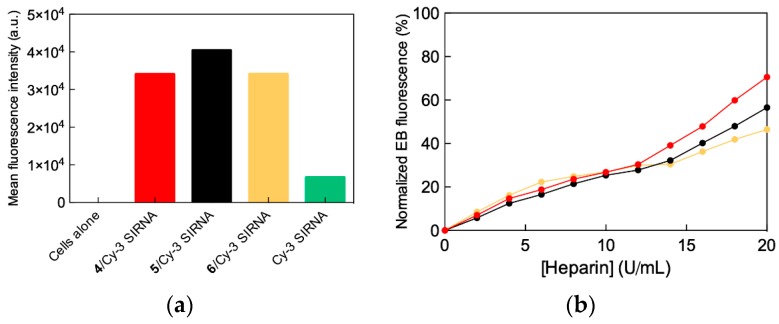
(**a**) The mean fluorescence intensity of PC-3 cells treated with siRNA labeled with the fluorescent dye Cy-3 in complex with self-assembled nanovectors **4**, **5**, and **6** (50 nM siRNA, N/P = 10). Non-treated cells and cells treated with labeled siRNA alone were used as control. (**b**) siRNA release from their complexes with micelles of dendrons **4** (red), **5** (black), and **6** (yellow) as assessed using EB displacement assay performed in the presence of heparin. Adapted from [7] with the permission of John Wiley and Sons, 2016.

These last results seem to be in slight contradiction with the computer-based prediction reported in Table 2, according to which the best (i.e., most favorable) values of ΔG_bind,eff_/N_eff_ and C_50_ are indeed obtained for self-assembled dendrons **5** and **6**. This ranks these nanovectors as the tightest siRNA binders. To investigate whether this disagreement was real or only apparent, cellular uptake experiments based on flow cytometry were performed using siRNA in complex with micelles from **4**, **5**, and **6** (Figure 5a). These data clearly show that this step is not governing the differential behavior of these nanovector/siRNA performance along the in vitro gene silencing pathway, as all three systems are characterized by comparable cellular uptake efficiency.

With this aspect ruled out, it was reasoned that the siRNA release of its nanovector could be the factor underlying the best performance in gene silencing of the **4**/siRNA complex. Thus, it was determined that it was the dissociation of the nucleic acid fragments from their **4**, **5**, and **6** self-assembled nanovectors via the EB displacement assay in the presence of heparin, a highly negatively charged polysaccharide that can compete with siRNA in nanocarrier binding. As can be seen in Figure 5b, by increasing heparin concentration the siRNA molecules were more efficiently displaced from their complex with **4** than from the two alternative dendron micelles. This led us to the conclusion that the siRNA release process might be more effective from the self-assembled dendron **4** than from the **5** and **6** complexes, respectively.

Collectively, these results led to the conclusion that, in agreement with computer predictions, the supermolecular assemblies formed by **5** and **6** were actually too stable to dissemble in physiological conditions. This enhanced stability negatively affects the siRNA release in the cellular environment, thereby impairing the relevant gene silencing effect. At the same time, the complexes of micelles originated by dendron **4** and siRNA represent the best compromise in term of nucleic acid fragment binding and release efficiency, and these properties ultimately translate into the most potent biological performance.

**Figure 6:**
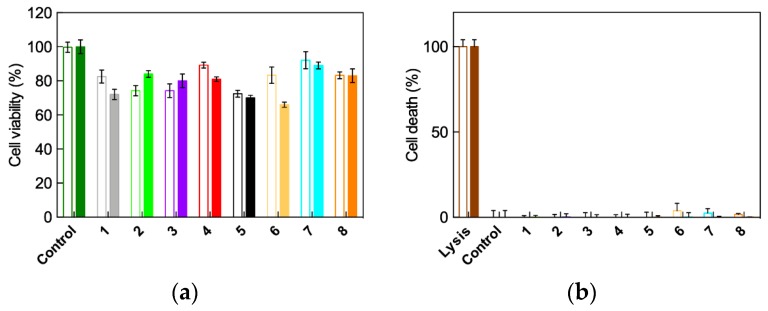
Metabolite toxicity (**a**) and membrane damaging activity (**b**) of self- and non-self-assembling dendrons **1**–**8** and their complexes with a scrambled (i.e., non-silencing) siRNA sequence (50 nM siRNA, N/P = 10) in PC-3 cells using the 3-(4,5-dimethylthiazol-2-yl)-2,5-diphenyl tetrazolium bromide (MTT) assay and the lactate dehydrogenase (LDH) assay, respectively. Untreated cells were used for control. Adapted from [7] with the permission of John Wiley and Sons, 2016.

The final step of the in vitro characterization concerned the investigation of the cellular toxicity of our single-tail self-assembled dendrons. As shown in Figure 6 for the PC-3 cell line as an example, none of the self- and non-self-assembling dendrons were endowed with cytotoxic properties. Analogous results were obtained with the alternative prostate cancer cell line, C4-2.

In essence, all in silico and in vitro results concurred to indicate **4** as the amphiphilic dendron endowed with the best features for efficient self-assembly, and easy and stable formation of complexes with the siRNA molecules for delivery. Once inside the cell, there is effective disassembly during endosomal release for subsequent potent gene silencing.

### 2.4. In Vivo siRNA Delivery Performance of Single-Tail Self-Assembling Dendron **4**

**Figure 7:**
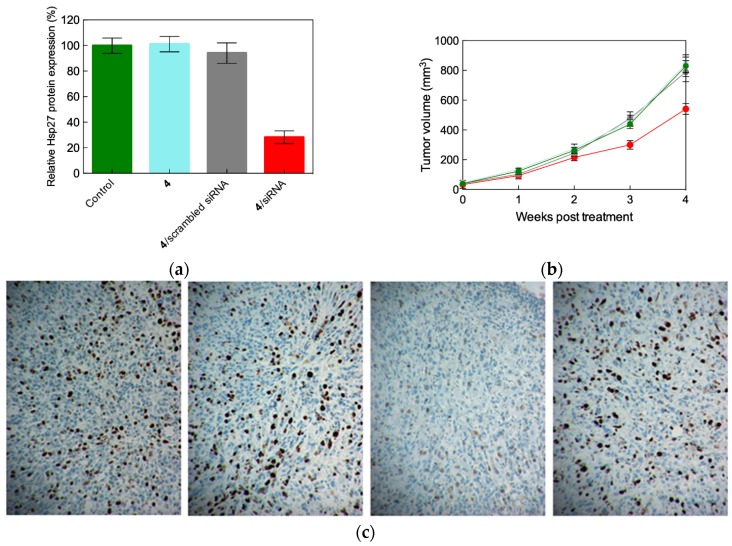
(**a**) In vivo gene silencing achieved by treating nude mice bearing prostate cancer (PC-3) tumors (30–50 mm^3^) with Hsp27 siRNA delivered by the self-assembling dendron **4**, buffer solution (control), the dendron **4** alone, and a scrambled siRNA/**4** complex (negative controls). Treatments (3 mg/kg siRNA, N/P = 5) were administered for a period of 4 weeks via intraperitoneal injections (twice per week). (**b**) In vivo inhibition of tumor growth quantified by tumor size and (**c**) antiproliferation activity in tumors assessed by immunohistochemistry in mice treated as described for panel (**a**). Panel (**c**), from left to right: control, **4** alone, **4**/siRNA, **4**/scrambled siRNA. Adapted from [7] with the permission of John Wiley and Sons, 2016.

The in vitro best performing nanomicelles generated by the dendron **4** were finally challenged for in vivo gene silencing in a prostate cancer xenografted nude mouse model. The results obtained are gathered in Figure 7.

Images in Figure 7 support the effective and specific in vivo gene silencing induced upon administration of the Hsp27-directed siRNA delivered by the self-assembled nanovector **4**. In particular, an impressive reduction of ~80% in Hsp27 expression and of ~65% in tumor size after 4 weeks of treatment were achieved (Figure 7a,b).

**Figure 8:**
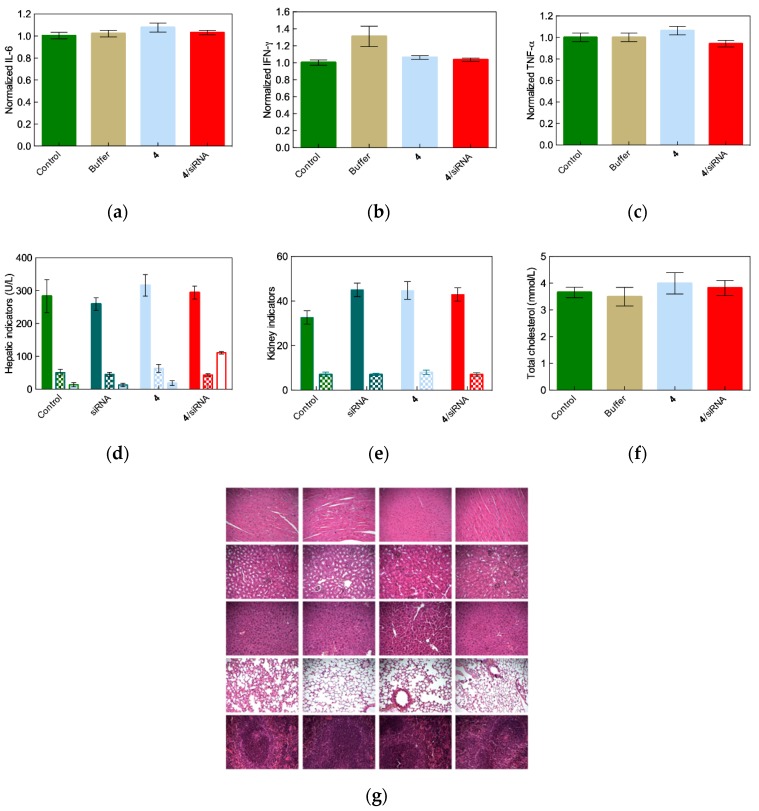
In vivo levels of inflammatory cytokines interleukine-6 (IL-6, **a**), interferon-gamma (INF-γ, **b**), and tumor necrosis factor-alpha (TNF-α, **c**), observed in male C57BL/6 mice treated with **4** and its siRNA complex (3.0 mg/kg siRNA, N/P = 5) by intravenous injection. Non-treated mice and animals treated with buffer solutions were used for control. Mice were sacrificed 24 h after treatment and the ELISA assay was performed on the collected serum. (**d**) Hepatic enzyme levels (aspartic aminotransferase AST (solid bars), alanine transferase ALT (patterned bars), gamma-glutamine transferase γ-GLT (empty bars), (**e**) kidney functions (creatinine CRE (mmol/L, solid bars), blood urea nitrogen BUN (μmol/L, patterned bars), and (**f**) cholesterol level in the blood measured in the treated mice after sacrifice. (**g**) Pathology of major organs from treated mice (HES staining). Columns (from left to right): Control, **4**, **4**/siRNA, and **4**/scrambled siRNA. Rows (from top to bottom): Heart, kidney, liver, lung, spleen. Adapted from [7] with the permission of John Wiley and Sons, 2016.

These data, coupled with no discernable signs of in vivo toxicity (Figure 8) and the absence of mice weight loss, support the potential utilization of the amphiphilic compound **4** as a siRNA vector for future therapeutic implementations.

## 3. siRNA Delivery by Double-Tail Self-Assembling Amphiphilic Dendrons

### 3.1. Design, Optimization and Chemico-Physical Charactrization of Double-Tail Self-Assembling Amphiphilic Dendrons

**Figure 9:**
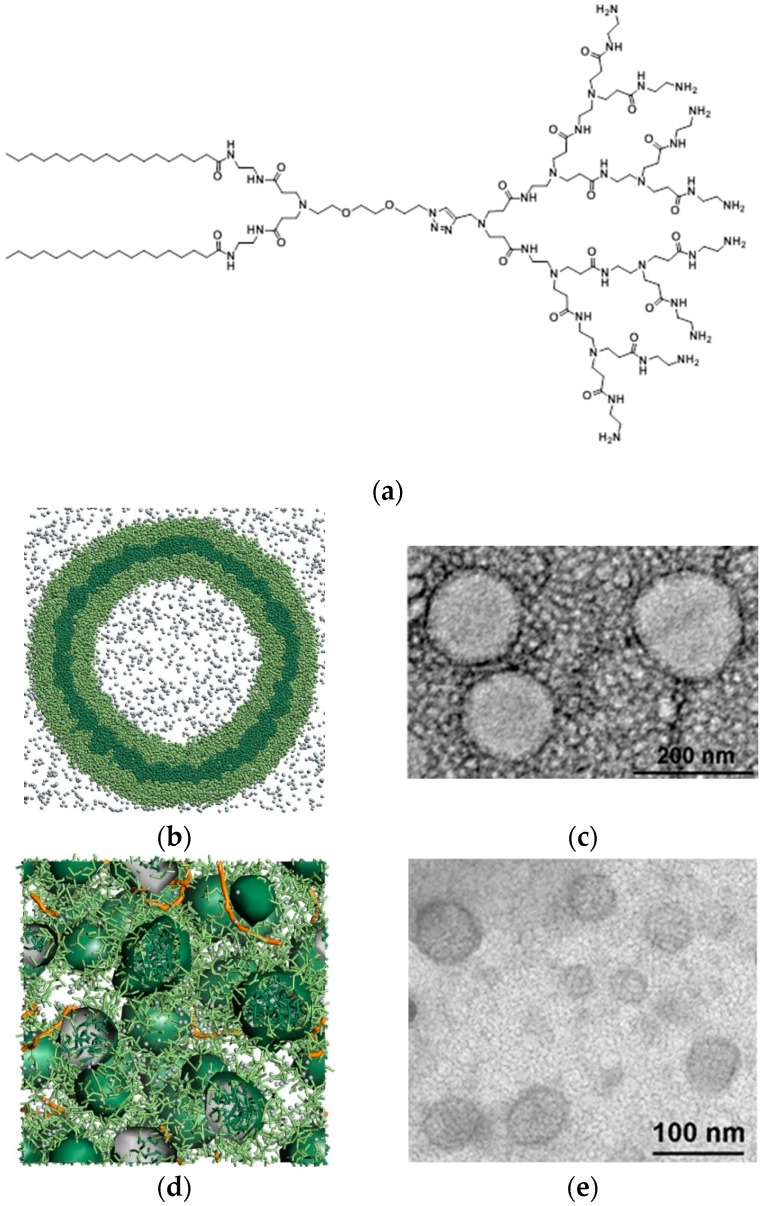
(**a**) The structure of the double-tail amphiphilic dendron simulated (**b**) and experimental TEM images (**c**) of the vesicle-like dendrimersomes formed by **AD** alone upon self-assembling in buffer solution. In panel b, the hydrophilic units of **AD** are portrayed in light green, the hydrophobic units are depicted in dark green, while light grey spheres are used to show some representative water molecules. Simulated (**d**) and experimental TEM images (**e**) of the nanosized spherical micelles formed by **AD** in the presence of siRNA molecules in solution. In panel (**d**), colors as in panel (**b**), while the nucleic acid fragments are portrayed as orange sticks. Adapted from [6] with permission of John Wiley and Sons, 2014.

With the haste of further improving the siRNA delivery and related gene silencing performance of the lead self-assembling amphiphilic dendron **4**, it was questioned whether increasing its hydrophobic portion would render this molecule even more efficient and effective. According to the devised strategy, it was ultimately decided to decorate **4** with a second C_18_-long alkyl chain. In order to do so, a series of computer-based molecular design and optimization studies were carried out, finally leading to the new double-tail amphiphilic dendron structure **AD** (where **AD** stands for Amphiphilic Dendron) shown in Figure 9a [6]. In buffer solution per se, molecular simulations predicted **AD** to self-assemble into large (~200 nm) vesicle-like structures (Figure 9b) called dendrimersomes [16]. After synthesis of **AD** by click-chemistry, dynamic light scattering (DLS) and transmission electron microscopy (TEM) indeed confirmed both the size and the shape of the supermolecular entities formed by **AD** upon self-assembly (Figure 9c). Notably, the vesicle corona structure closely matched a bilayer, characterized by a thickness of 7 nm—approximately equal to twice the molecular length of **AD** (~3.5 nm).

Quite surprisingly, however, when simulations of the self-assembly of **AD** were performed in the presence of siRNA many highly ordered smaller spherical micelles of 6–8 nm diameter were obtained, with the siRNA fragments nicely interspersed within them (Figure 9d). The explanation for this vesicular to micellar structural transition likely resides in the greater positively charged surface area exposed by the micelles with respect to the dendrimersomes, which provides more efficient electrostatic interactions with the negatively charged siRNA. This, in turn, leads to the formation of more stable and compact complexes between the self-assembled nanocarriers and the nucleic acids molecules. The computer predictions were supported by TEM imaging (Figure 9e), confirming that **AD** is able to dynamically self-assemble into responsive and adaptive supramolecular assemblies in the presence of external stimuli, i.e., negatively-charged small polyanions.

### 3.2. In Vitro siRNA Delivery Performance of the Double-Tail Self-Assembling Dendron AD

**Figure 10:**
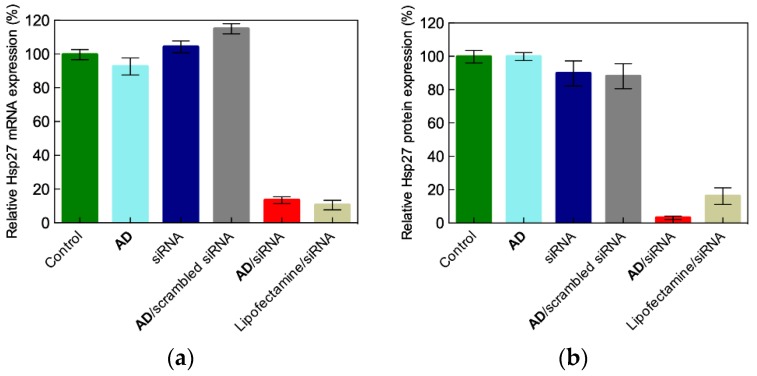

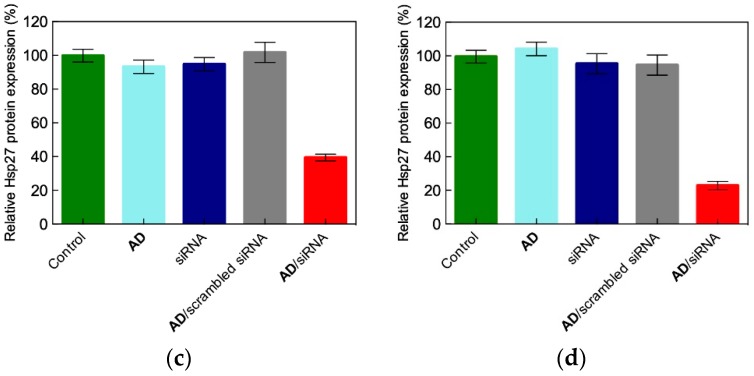
Downregulation of Hsp27 at the mRNA (**a**) and protein (b) level in prostate cancer PC-3 cells treated with siRNA (50 nM) delivered by the self-assembled double-tail dendron **AD** (N/P = 5). Inhibition of Hsp27 expression by siRNA/**AD** complexes (50 nM siRNA, N/P = 5) in breast cancer MCF-7 (**c**) and MDA-MB231 (**d**). Adapted from [6] with permission of John Wiley and Sons, 2014.

The nanosized micelles formed by self-assembly of the double-tail amphiphilic dendron **AD** were able to effectively protect siRNA from degradation (e.g., by RNAses) and to promote fast and quantitative uptake by castrate-resistant prostate cancer PC-3 cells—almost 100% internalization of the nanovector/siRNA complexes was reached within 30 min. As concerns the mechanism of cell internalization of the **AD**/siRNA complexes, macropinocytosis was found to be the leading cellular entry mechanism for this nanovector/siRNA system, in utter analogy with the G_5_ covalent PAMAM dendrimer counterpart discussed in the companion paper (Figure 15a in [9]). Indeed, neither chlorpromazine (an inhibitor of clathrin-mediated endocytosis) nor genistein (a caveolae-mediated uptake specific blocker) were effective while cytochalasin D (a specific macropinocytosis inhibitor that, by binding to actin filaments, interferes with actin polymerization and assembly) inhibited cellular uptake in a dose-dependent manner, as presented in Figure 10b. Again in analogy with what observed for the G_5_ TEA-core PAMAM covalent dendrimer (Figure 15b in [9]), confocal microscopy provided images showing significant co-localization of the siRNA/**AD** complexes with the macropinocytosis biomarker dextran. Whereas, negligible co-localization was observed in cells treated with the two alternative biomarkers transferrin (clathrin-mediated endocytosis) and CTX-B (caveolae-mediated endocytosis). Thus, these experiments further corroborate the concept that these self-assembling PAMAM-based amphiphilic dendrons behave very similarly to their covalent high generation counterpart.

The gene silencing effect induced by Hsp-27 targeting siRNA delivered by **AD** nanomicelles was next investigated both at the mRNA and protein levels using prostate (PC-3) and breast (MCF-7 and MDA-MB231) cancer cell lines. As seen in Figure 10, a remarkable attenuation of the Hsp27 expression was achieved in all cases. This effect was comparable, if not greater, than that achieved with Lipofectamine, the reference standard reagent for siRNA/DNA transfection (e.g., **AD** was 6 times more efficient than Lipofectamine in downregulating Hsp27 protein expression in PC-3 cells, Figure 10b).

The knockdown of Hsp27 in these cancer cells by siRNA delivered by **AD** micelles was paralleled by significant anti-proliferative effects (~75% of cancer cell growth inhibition), in agreement with the evidence that Hsp27 silencing negatively affects PC-3 cell survival [9,17]. Similarly to what was observed for the high generation covalent PAMAM dendrimers [9], the mechanism leading to cell death relied on caspase-dependent induced apoptosis, that is, the programmed cell death was promoted by apoptotic caspases, a family of endoproteases that provide critical links in cell regulatory networks controlling cell death. Accordingly, a 3-fold increase in caspase-3/7 activation was observed on average, confirming that siRNA delivered by the nanovectors based on self-assembled **AD** is very effective in silencing Hsp27 and thereby inducing caspase-dependent anticancer activity in vitro. A remarkable additional feature of this biological outcome is that the silencing effect was completely maintained for one week, and was also effective when transfection was performed in the presence of serum, a prototypical Achilles’ heel of cationic nanovectors in siRNA delivery. In fact, the negatively charged serum proteins can not only compete with siRNA binding to the nanocarrier, but also may, by virtue of strong electrostatic forces, lead to the the disintegration of the nanoassemblies with consequent premature nucleic acid release and degradation [9].

**Figure 11:**
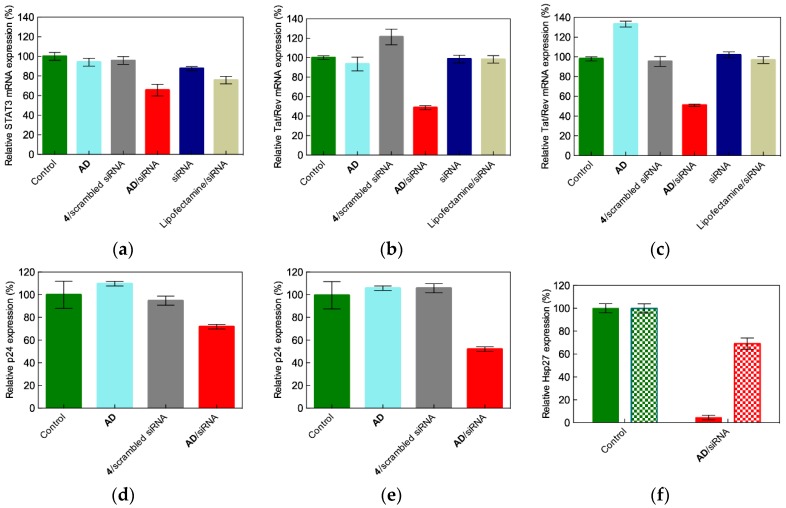
(**a**) STAT3 knockdown in glioblastoma stem cells (PBT003) treated with siRNA/**AD** complexes (50 nM siRNA, N/P = 5). The knockdown of Tat/Rev in primary human peripheral blood mononuclear cells (PBMC-CD4^+^) (**b**) and hematopoietic CD34^+^ stem cells (HSC-CD4^+^) (**c**) treated as in (**a**). Effective inhibition of HIV replication in PBMC-CD4^+^ (**d**) and HSC-CD4^+^ (**e**) after treatment with the anti-Tat/Rev siRNA delivered by **AD** (50 nM siRNA, N/P = 5). Viral loading was assessed using HIV-1 p24 antigen ELISA 3 days post-treatment. PBMC-CD4^+^ cells and HSC-CD34^+^ cells were infected by NL4-3 virus at a multiplicity of infection (MOI) = 0.001 and by JR-FL virus (MOI = 0.005), respectively, for 5 days before transfection. (**f**) Proton-sponge effect in PC-3 cells transfected with siRNA/**AD** in the absence (solid bars) and presence (patterned bars) of the proton pump inhibitor Bafilomycin A1. Untreated cells were used as the control. Adapted from [6] with permission of John Wiley and Sons, 2014.

From another perspective, when confronted with its single-tail analogue **4,** the efficiency of in vitro silencing induced by the double-tailed **AD** dendron/siRNA complexes was practically identical (e.g., 97% for **AD** and 96% for **4** in PC-3 cells, Figure 10b,c, respectively). Most importantly, however, remarkably good results were obtained with **AD** when challenged against cancer stem cells (notoriously the cancer cell population most refractory to treatments), and also stem and primary cells that constitute the target of deadly viruses such as HIV-1. As shown in Figure 11, **AD** was indeed able to elicit significant RNAi interference (RNAi) in glioblastoma stem cells (PBT003), and in HIV-1 infected human primary peripheral blood mononuclear cells (PBMC-CD4^+^), and hematopoietic stem cells (HSC-CD34^+^). Specifically, upon treatment of the hardly tractable PBT003 cells with **AD** carrying the siRNA was directed against the signal transducer and activator of transcription 3 (STAT3), which is a key player protein in glioma-initiating cells, thought to be responsible for glioblastoma induction, progression and recurrence. A 35% reduction in the corresponding mRNA level was observed, while only 24% mRNA reduction was achieved upon transfection with Lipofectamine (Figure 11a). The data relative to the two HIV-infected cell lines (PBMC-CD4^+^ and HSC-CD34^+^) transfected with the siRNA targeting the HIV-1 Tat/Rev gene are more striking. The Tat and Rev HIV-1 proteins are essential positive regulators of gene expression through interaction with RNA target elements present within the 5′ translated leader sequence and envelope gene, respectively. Importantly, the genes encode the Rev and Tat overlap, with each being produced from a different reading frame. As seen from Figure 11b,c, in both cases a remarkable 50% reduction in the Tat/Rev mRNA expression was observed, while the prototypical delivery vector Lipofectamine (aka RNAiMAX) failed to induce any gene silencing in these cells infected by the acquired immunodeficiency syndrome (AIDS) virus. Fundamentally, this translated into a 50% reduction of the viral infection (Figure 11d,e) and, to the best of the authors’ knowledge, constitutes the first report on the successful and safe delivery of siRNA into primary and stem cells.

As for the high generation covalent PAMAM dendrimer analogues presented in the companion paper [9], it was reasoned that also in the case of the **AD** micelles, the so-called proton sponge effect [18] could be invoked to explain nucleic acid release from the self-assembled double-tail nanovectors and, therefore, potent siRNA delivery within the cell cytoplasm. According to the debated proton sponge concept, when cationic nanoparticles such as those formed by **AD** and its siRNA cargo enter acidic endosomal (and lysosomal) vesicles, unsaturated amino groups sequester protons supplied by the proton pump v-ATPase. These sequestered protons cause the pump to continue functioning, leading to the retention of chloride ions and water molecules. Eventually, osmotic swelling causes the rupture of the vesicle, allowing the cationic nanoparticles to enter the cytoplasm. As the **AD** amphiphiles feature tertiary amines in the interior of their PAMAM heads which become protonated at the endosomal acidic pH (5.5), the authors verified whether the proton sponge effect was effectively at play also during their mediated siRNA delivery. Indeed, as illustrated in Figure 11f, Hsp27 silencing in PC-3 cells was significantly reduced in the presence of Bafilomycin A1 (a proton pump inhibitor that prevents endosome acidification). This implies that the **AD**-mediated siRNA delivery was indeed dependent on the endosomal acidification process and that the proton sponge effect played a role in the endosomal escape and cytoplasmic release of the nucleic acid.

Finally, the same protocol adopted for investigating the cellular toxicity of the single-tail self-assembled dendrons described in Section 2.3 was applied to **AD**. As expected, no discernible toxicity was observed in all cell lines (PC-3, PBMC-CD4^+^, HSC-CD34^+^, and PBT003 transfected with 50 nM siRNA and **AD** at N/P = 5 and 10) using both the MTT and LDH assays, supporting further in vivo gene silencing experiments with this nanocarrier.

### 3.3. In Vivo siRNA Delivery Performance of the Double-Tail Self-Assembling Dendron **AD**

**Figure 12:**
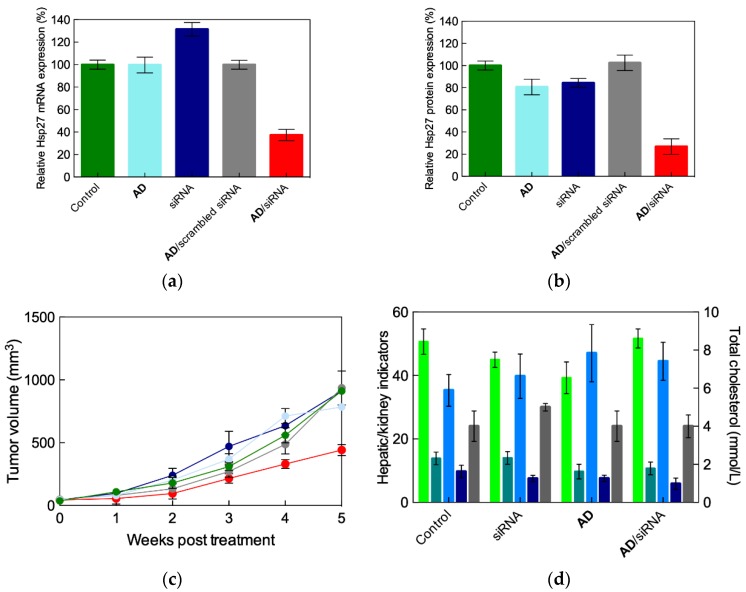
In vivo effective gene silencing of Hsp27 at the mRNA (**a**) and protein (**b**) levels observed in prostate cancer (PC-3) xenograft mice after treatment with Hsp27 siRNA delivered by **AD** (3 mg/kg siRNA and **AD** at N/P = 5, injection twice a week for 5 weeks). Hsp27 mRNA and protein levels were quantified by quantitative reverse transcription polymerase chain reaction (qRT-PCR) and western blotting, respectively. Non-treated mice were used for the control. **AD** alone, siRNA alone and a scrambled (non-silencing) siRNA sequence delivered by **AD** were used as negative controls. (**c**) Tumor volume during treatment of PC-3 mice xenografts with siRNA/**AD** complexes (red symbols), compared with non-treated animals (green), and animals treated by AD alone (light blue), siRNA alone (dark blue), and a scrambled (non-silencing) siRNA sequence delivered by **AD** (gray symbol) used as negative controls. (**d**) Hepatic enzyme levels (alanine transferase ALT (light green bars), gamma-glutamine transferase γ-GLT (teal bars), (kidney functions (creatinine CRE (mmol/L, sky blue bars), blood urea nitrogen BUN (μmol/L, dark blue bars), and cholesterol level in the blood (gray bars, values on the right axis) measured in male C57BL/6 mice treated with intravenous administration of siRNA/AD (3 mg/kg siRNA and **AD** at N/P = 5). Untreated mice were used for control. Adapted from [6] with permission of John Wiley and Sons, 2014.

**Figure A2:**
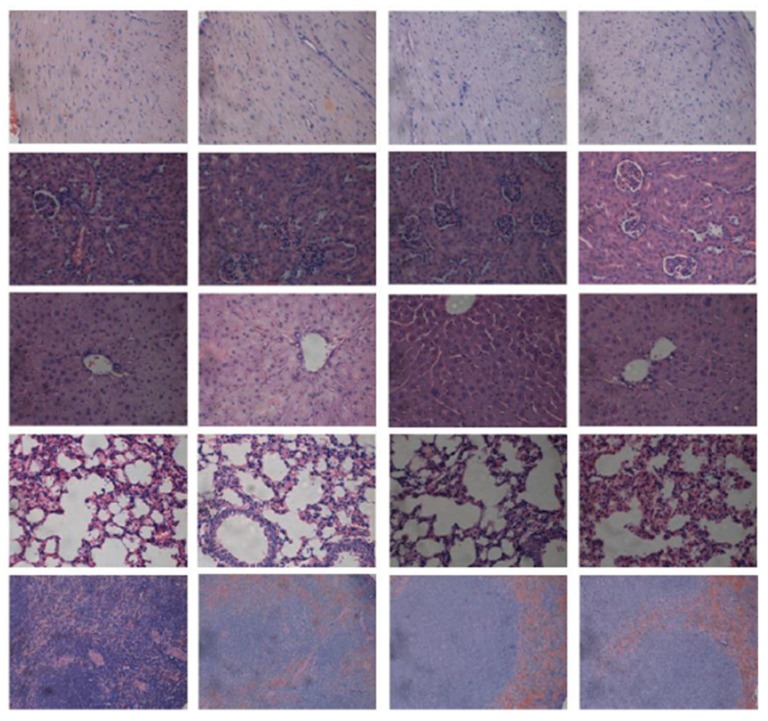
Pathology of major organs from treated mice (HES staining). Columns (from left to right): control, siRNA, **AD**, **AD**/siRNA. Rows (from to bottom): heart, kidney, liver, lung, spleen. Adapted from [6] with the permission of John Wiley and Sons, 2014.

The final steps to assess the therapeutic potential of siRNA delivery based on the double-tail self-assembled amphiphilic dendron **AD** were constituted by in vivo experiments using prostate cancer (PC-3) xenograft mouse models and Hsp27 as the target gene for knockdown. Figure 12a,b reveal that, following a 5-week animal treatment by intraperitoneal injections (2/week) of siRNA/**AD** complexes (3 mg/kg Hsp27 siRNA, **AD** at N/P = 5), there was a remarkable downregulation of Hsp27 at both mRNA (62%) and protein (73%) levels. This was paralleled by an effective inhibition of tumor growth, as shown in Figure 12c. Further, as for the case of the single-tail amphiphilic analogue **4**, no major organ injury or histopathological changes were evidenced in the animal subjected to **AD**/siRNA treatments (Figure 12d and Figure A2).

In summary, the nanosized micelles generated upon self-assembly of the double-tail amphiphilic PAMAM-based dendron **AD** were not able to deliver siRNA to a different panel of cancer cell lines with efficiency in vitro and in vivo comparable to those formed by its single-tail precursor **4**. However, they were also capable of eliciting gene silencing effects in the highly challenging and treatment-refectory human primary cells and stem cells. Accordingly, these versatile and safe delivery properties of **AD**, coupled with easy formulation, render AD a promising nanocarrier for highly efficient, cell-type-independent RNAi therapeutics.

## 4. siRNA Delivery by the Double-Tail, Dual Targeting Self-Assembling Amphiphilic Dendron AD/E_16_G_6_RGDK

### 4.1. Design, Optimization and Chemico-Physical Charactrization of the Double-Tail, Dual Targeting Self-Assembling Amphiphilic Dendron **AD**/E_16_G_6_RGDK

In the general design strategy of nanocarriers to achieve effective siRNA (and in general drug) delivery, two well-known criteria must be satisfied: (i) The delivered therapeutic is be able to reach the desired disease (e.g., tumor) site(s) after administration with minimal loss in their quantity and activity during their journey in the blood stream; and (ii) they only affect diseased cells without exerting harmful effects to healthy organs and tissues. These requirements may be enabled using two strategies: Passive and active drug targeting of drugs. The former approach relies on the (still controversial) intrinsic enhanced permeability and retention effect (EPR) [19,20], according to which molecules with size in the nanometer range tend to accumulate in diseased (especially tumor) tissues much more than they do in normal tissues, thereby leaving little space for specific molecular design and optimization. Active targeting employs directed intermolecular interactions (e.g., ligand binding to disease-overexpressed receptors or other molecular recognition processes) to confer more specificity to the delivery system. In the end, besides enhanced therapeutic action, active targeting can result in reduced non-specific interactions and localization of the drug in peripheral tissues, thus minimizing unwanted side-effects. One of the biggest challenges in active targeting consists in designing new nanovectors (or modifying existing performing ones) with targeting moieties that do not interfere with its cargo loading capacity and its final mission purposes. Obviously, these chemical entities must be fully available to interact with their cellular counterparts and be endowed with strong affinity towards their targets (e.g., surface receptors) in order to trigger endocytosis.

With the goal of empowering the high-performance double-tail self-assembling amphiphilic dendron **AD** with active targeting ability for siRNA specific delivery to cancer cells, the authors considered the RGDK peptide as the starting point for molecular design and optimization. The RGDK peptide is an ideal moiety for cancer targeted nanomedicine in that it features a dual targeting capacity within a single, small molecular scaffold. On the one side, the RGD sequence is known to be able to target tumor endothelium via interaction with ανβ_3_-integrin (a receptor overexpressed in tumor vasculature [21]); while on the other side, the RGDK sequence has been shown to bind the neutropilin-1 (Nrp-1, a transmembrane hub receptor with multiple ligands that is abundant on the surface of cancer cells [22]), and in so doing, promoted cell uptake and penetration.

**Figure 13:**
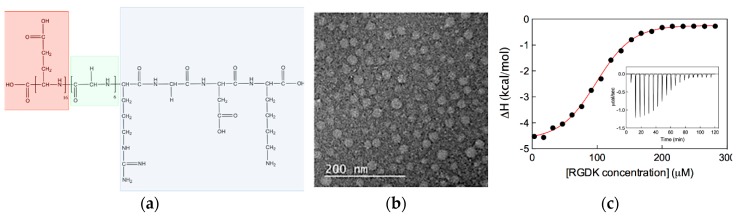
(**a**) Chemical structure of the E_16_G_6_RGDK peptide designed as a double targeting moiety for enhancing the siRNA delivery to cancer cells mediated by the double-tail self-assembling **AD** dendron. The light red box encases the E_16_ fragment, the light green box marks the G_6_ spacer while the light blue box highlights the RGDK warhead. (**b**) TEM imaging of the siRNA/**AD**/ E_16_G_6_RGDK nanosized micelles formed upon simple addition of the targeting peptide to pre-formed siRNA/**AD** complexes. (**c**) Integrated ITC profile for the titration of siRNA/**AD** complexes with the E_16_G_6_RGDK peptide. The solid red line represents data fitting with a sigmoidal function. The insert shows the corresponding ITC raw data. Adapted from [8] with permission of the American Chemical Society, 2018.

Several sequence rounds of computer optimization led to the final design of the E_16_G_6_RGDK peptide shown in Figure 13a [8]. This new chemical entity features three distinct segments serving different functions, as follows: (1) A highly negatively charged tail composed of 16 glutamic acid residues (E_16_), for anchoring the peptide to the positively charged surface of the self-assembled **AD** micelles via strong, favorable electrostatic interactions; (2) the positively charged RGDK warhead for fostering cancer cell targeting and homing by virtue of the dual interaction with ανβ_3_-integrin and Nrp-1 receptors; and (3) a neutral spacer of optimal length composed of 6 glycine residues (G_6_) separating the oppositely charged segments, (1) and (2).

With the optimized new peptide structure at hand, and the previous knowledge about the self-assembling process of **AD** and its related performance as siRNA nanocarrier (Section 3), the authors initially verified the interaction of the E_16_G_6_RGDK molecules with pre-formed siRNA/**AD** complexes in solution by means of different experimental techniques. Both DLS and TEM confirmed the formation of nanosized (30–45 nm in diameter) spherical micelles (shown in Figure 13b), characterized by a ζ-potential value of +15 mV, approximately equal to half of the value measured for the siRNA/**AD** assemblies (+32 mV). This substantial decrease in positive surface charges further supported the interaction of the negatively charged (−15) peptide with the siRNA/**AD** complexes.

However, the ultimate confirmation of the effective binding of the E_16_G_6_RGDK targeting peptides to the siRNA/AD nanomicelles was obtained by isothermal titration calorimetry (ITC). ITC is a straightforward and noninvasive titration-based method for binding interaction analysis performed in solution at constant temperature [23]. The analysis of ITC curves directly yields the binding enthalpy (ΔH_bind_) and, upon data fitting with a suitable thermodynamic model, the binding constant K_b_ can be obtained. Once K_b_ is known, the free energy of binding ΔG_bind_ is calculated using the relationship ΔG_bind_ = −RT lnK_bind_, where R is the universal gas constant and T is the absolute temperature. Finally, the variation in entropy upon binding is derived from the fundamental Gibbs equation ΔG_bind_ = ΔH_bind_ − TΔS_bind_. The binding thermodynamics between the targeting peptides and the siRNA/AD micelles was found to be characterized by a favorable (i.e., negative) enthalpic variation (ΔH_bind_ = −5.0 ± 0.2 kcal/mol), as evidenced by the exothermic peaks in the corresponding thermogram (Figure 13c). Also, a small, favorable (i.e., positive) entropic change (TΔS = 1.9 kcal/mol) was estimated, leading to an overall favorable (i.e., negative) ΔG_bind_ value of −6.8 kcal/mol. The substantial enthalpic nature of the binding between the RGKD peptide and the siRNA/**AD** micelles is the result of electrostatic forces as the main supramolecular interaction drivers. However, the favorable entropic contribution was ascribed to the stabilizing peptide/micelle hydrophobic interactions and the concomitant release of ions and water molecules into the bulk solvent [24,25,26].

**Figure 14:**
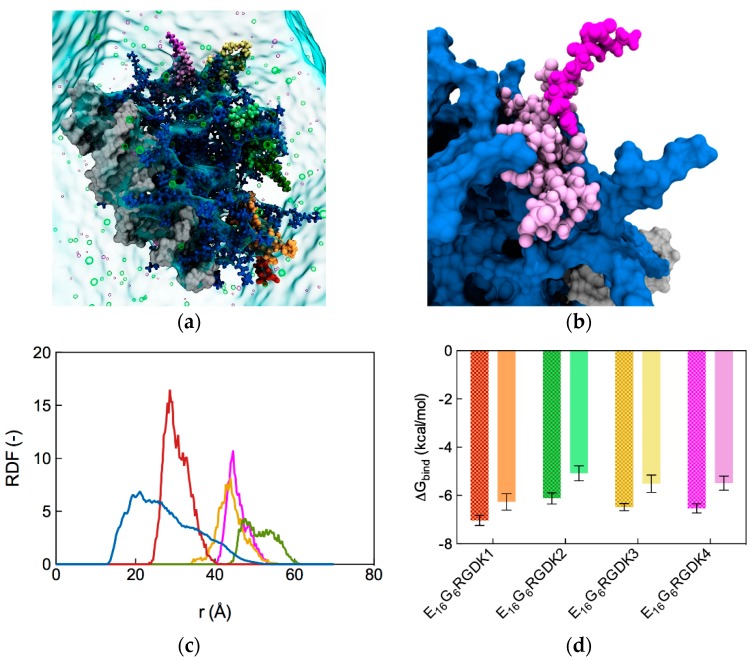
(**a**) Image extracted from the equilibrated portion of the MD simulation of a siRNA/**AD** micelle in complex with 4 E_16_G_6_RGDK peptides performed in 150 nM NaCl water solution. The 4 peptides are numbered and colored as follows: E_16_G_6_RGDK1, tail (E_16_G_6_) sandy brown, head (RDGK) firebrick; E_16_G_6_RGDK2, tail light green, head olive drab; E_16_G_6_RGDK3, tail khaki, head golden rod; E_16_G_6_RGDK4, tail plum, head magenta. The **AD** micelle is in a dark slate blue sticks-and-balls representation, the siRNA molecule is highlighted by its gray van der Waals surface, the peptides are shown as colored spheres, Na^+^ and Cl^−^ ions are depicted as purple and green hollow spheres, while a light cyan transparent surface is used to represent the water solvent. (**b**) Zoomed view of the image in panel (**a**), showing the E_16_G_6_RGDK4 peptide with its tail (plum) attached to the micelle surface and its RGDK warhead (magenta) protruding into the solvent. In this panel, water and ions are omitted for clarity. (**c**) Radial distribution function (RDF) from the **AD** center of mass for the positively charged **AD** terminal groups (dark slate blue), and for each of the four RGDK warhead groups (colors as in panel (**a**)). (**d**) Free energy of binding (ΔG_bind_) of each E_16_G_6_RGDK peptide on the siRNA/**AD** micelle. The patterned bars represent the ΔG_bind_ values for the entire peptide molecule, while the solid bars show the contribution to ΔG_bind_ afforded by the negatively charged (E_16_G_6_) tails only. Adapted from [8] with permission of the American Chemical Society, 2018.

The structure of the siRNA/**AD**/E_16_G_6_RGDK was next investigated at the molecular level via computer simulations. Extensive (1 μs) atomistic molecular dynamics (MD) simulations revealed the formation of stable, Janus-like nanoparticles featuring a siRNA molecule bound on one face of each **AD** micelle and 4 peptides adsorbed on the opposite face (Figure 14a). Further, MD simulations showed that the negatively charged peptide tails (E_16_G_6_) were adsorbed onto the positively charged surface micelle while, most importantly, the 4 RGDK warhead was free from sterical hindrance and protruded deeply into the solvent (Figure 14b).

These pictorial views were quantified first by calculating the radial distribution function (RDF) from the **AD** micelle center of mass for the positively charged **AD** terminal groups and the 4 targeting peptide RGDK warheads, as shown in Figure 14c. As seen from this Figure, all 4 RGDK moieties are positioned far away from the nanomicelle periphery. Accordingly, they are potentially available for interaction with their cellular target receptors. Next, the free energy of binding ΔG_bind_ of each single E_16_G_6_RGDK peptide with the siRNA/**AD** nanomicelle was estimated by processing the MD data according to the MM/PBSA methodology [9,10,11,12,13,14,15]. Figure 14d shows not only that all peptides are characterized by favorable ΔG_bind_ values (from −7.0 to −6.2 kcal/mol), but also that the major contribution to binding is afforded by the strong electrostatic interaction between the negatively charged peptide tails (E_16_G_6_) and the positively charged micellar surface, as expected by the original molecular design.

### 4.2. In Vitro Targeted siRNA Delivery Performance of Double-Tail, Dual Targeting Self-Assembling Amphiphilic Dendron AD/E_16_G_6_RGDK

**Figure 15:**
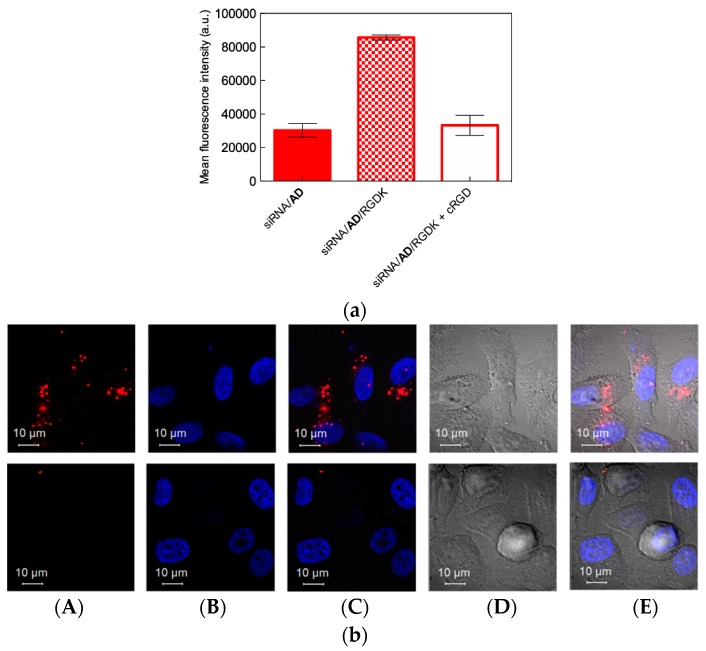
(**a**) Flow cytometry analysis of cellular uptake of siRNA/**AD** and siRNA/**AD**/E_16_G_6_RGDK nanoparticles in PC-3 cells using a siRNA labeled with the fluorescent Cy5 die. The last column shows the cell uptake results for the siRNA/**AD**/E_16_G_6_RGDK obtained in the presence of the cyclic peptide c-RGD, an ανβ_3_-integrin integrin binder. (**b**) Cellular uptake visualized by confocal microscopy of the siRNA/**AD**/E_16_G_6_RGDK complexes in the absence (top panel) and in the presence (bottom panel) of a Nrp-1 antibody. Images from left to right: (**A**) cy5-labelled siRNA; (**B**) nucleus (4′,6-diamidino-2-phenylindole (DAPI) staining); (**C**) merged (**A**,**B**). The last two images (**D**,**E**) are the bright field version of (**B**,**C**). In all these experiments, the siRNA/**AD**/E_16_G_6_RGDK nanoassemblies were prepared using 50 nM siRNA, siRNA/**AD** N/P = 10, and **AD**/E_16_G_6_RGDK molar ratio = 5. Adapted from [8] with permission of the American Chemical Society, 2018.

The authors verified that the presence of the E_16_G_6_RGDK peptides on the surface of the siRNA/**AD** micelles was indeed effective in specifically targeting cancer cells via dual binding with the designated target receptors ανβ_3_integrin and Nrp-1. The cellular uptake of the siRNA/**AD**/E_16_G_6_RGDK complexes was investigated again with PC-3 cells, in which both receptors were highly expressed [27,28]. As seen in Figure 15a, the amount of siRNA/**AD**/E_16_G_6_RGDK complexes internalized by PC-3 cells was three times higher than that measured for the siRNA/**AD** micelle. Further, the cellular uptake of the nanocarrier modified with the targeting moieties was inhibited in the presence of a cyclic RGD peptide. This clearly indicates that this last molecule competed with the peptide-coated nanovector for integrin binding via the RDG motif.

Next, PC-3 cells pretreated with a monoclonal antibody (mAb) directed against Nrp-1 were used to assay the internalization of the siRNA/**AD**/E_16_G_6_RGDK nanoassemblies in comparison with untreated cells. As can be easily seen in the corresponding confocal microscopy images (Figure 15b), in the presence of the Nrp-1 mAb (bottom panel), the uptake of the siRNA-loaded peptide-modified **AD** micelles was drastically reduced when the mAb was absent (upper panel). This indicates that the targeting peptide-decorated siRNA delivery system was effectively cell internalized also via its interaction of its RGDK warhead with the Nrp-1 receptor.

**Figure A3:**
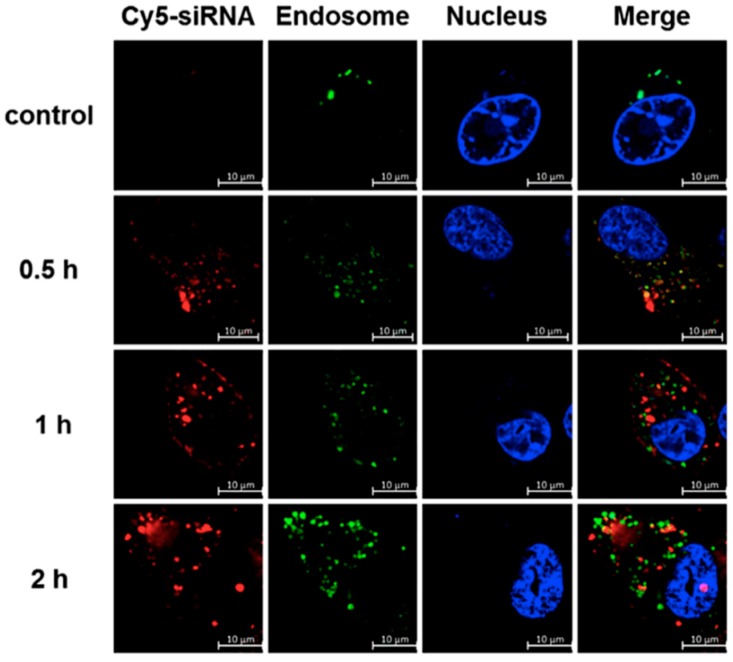
Confocal microscopy images of the successful endosomal escape of the siRNA/**AD**/E_16_G_6_RGDK nanoparticles in PC-3 cells after incubation times 0 (control), 0.5, 1, and 2 h Red channel images show the cy5-labeled siRNA/AD/E_16_G_6_RGDK nanoparticles, the green channel images reveal the Lyso Tracker red-marked endosomes, and the blue channel images show the cell nuclei stained with Hoechst 33342. In all these experiments, the siRNA/**AD**/E_16_G_6_RGDK nanoassemblies were prepared using 50 nM siRNA, siRNA/**AD** N/P = 10, and **AD**/E_16_G_6_RGDK molar ratio = 5. Adapted from [8] with the permission of the American Chemical Society, 2018.

Following verification of the successful attribution of the desired targeting properties to our double-tail self-assembling **AD** nanovector, the authors checked whether its excellent endosomal escape features, required for efficient siRNA delivery and release, was preserved. By confocal microscopy, it was observed that 2 h after transfection, almost all of the siRNA molecules were localized in the cell cytoplasm (Figure A3). This confirms that the peptide-decorated **AD** nanovectors were also able to effectively circumvent endosomal trapping and release their nucleic acid cargo for subsequent RNAi.

**Figure 16:**
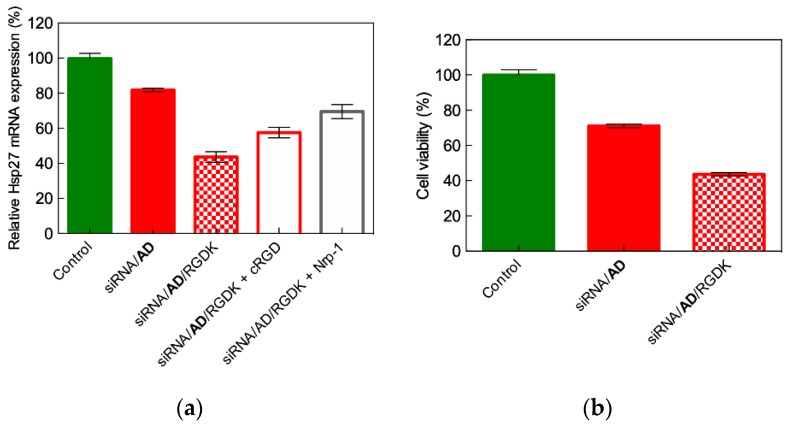
(**a**) mRNA levels following knockdown of Hsp27 in PC-3 cells with siRNA/**AD** and siRNA/**AD**/E_16_G_6_RGDK nanoparticles. The last two columns show the results of the same experiments performed in the presence of the integrin binder c-RGD and of the mAb directed toward the Nrp-1 receptor, respectively. (**b**) Comparison of the antiproliferative activity exerted on PC-3 cells by siRNA/**AD** and siRNA/**AD**/E_16_G_6_RGDK nanoparticles. In both panels, non-treated cells were used for control, siRNA concentration = 20 nM, siRNA/**AD** N/P = 10, and **AD**/E_16_G_6_RGDK molar ratio = 5. Adapted from [8] with permission of the American Chemical Society, 2018.

The authors further assessed whether the endowment of the double-tail self-assembling dendron **AD** with targeting properties eventually conferred by the RGDK-based peptides rendered this nanovector more effective in siRNA delivery to cancer cells with respect to the undecorated **AD** micelles. Their RNAi performance in silencing Hsp27 in PC-3 cancer cells was compared. The relevant results showed that a remarkable Hsp27 knockdown was achieved both at the mRNA (56%, Figure 16a) and the protein (90%) levels with a very low (20 nM) siRNA concentration (for comparison, a 50 nM siRNA concentration was required to produce similar results either with the covalent G_5_ PAMAM dendrimer [9] or with the **AD** micelles without targeting peptide decoration (Figure 10)). Moreover, the same experiments performed in the presence of either the c-RGD peptide or the Nrp-1 directed mAb resulted in a decreased gene silencing effect (as shown by the last two columns in Figure 16a). This confirms that the siRNA/AD/E_16_G_6_RGDK nanoparticles indeed interact with the two target receptors.

**Figure A4:**
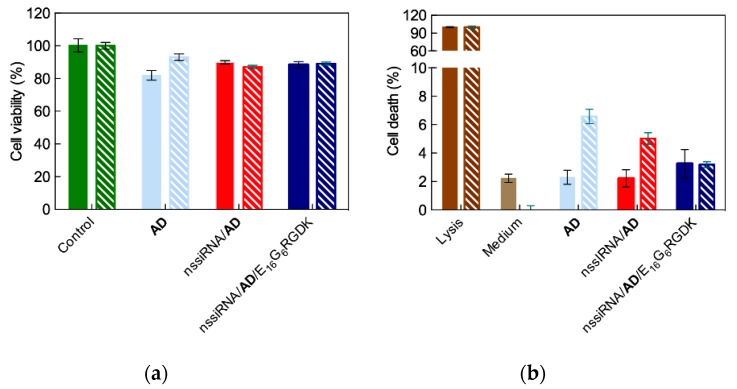

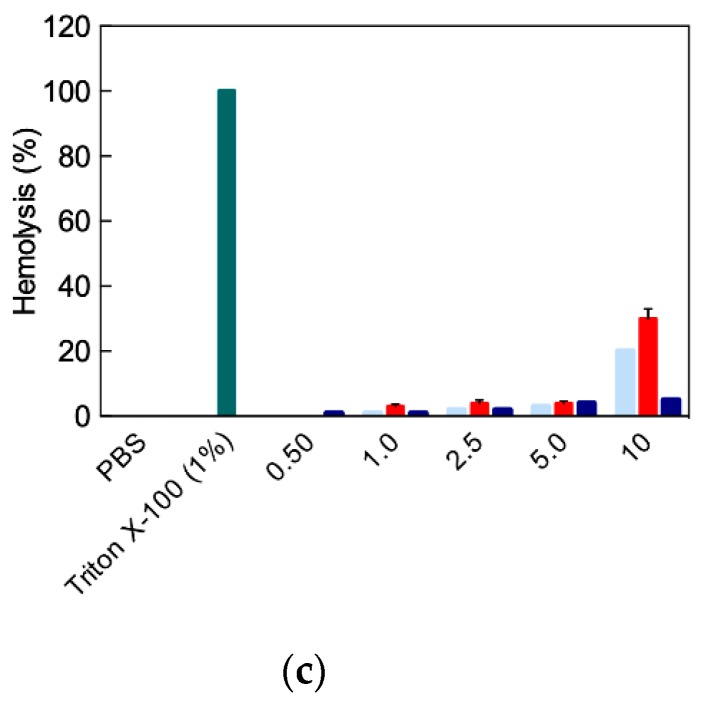
(**a**) Toxicity assessment using the MTT assay in prostate cancer PC-3 cells (solid bars) and in human embryonic kidney (HEK293) cells (patterned bars) of the **AD** micelles alone, **AD** in complex with a non-silencing siRNA (nssiRNA/**AD**) and the targeting peptide-decorated **AD** in complex with a non-silencing siRNA (nssiRNA/**AD**/E_16_G_6_RGDK). (**b**) Toxicity assessment using the LDH assay in prostate cancer PC-3 cells (solid bars) and in human embryonic kidney (HEK293) cells (patterned bars) of the **AD** micelles alone, **AD** in complex with a non-silencing siRNA (nssiRNA/**AD**) and the targeting peptide-decorated **AD** in complex with a non-silencing siRNA (nssiRNA/**AD**/E_16_G_6_RGDK). (**c**) Hemolysis effect measured via UV absorption at 540 nm for the **AD** micelles alone, **AD** in complex with a non-silencing siRNA (nssiRNA/**AD**) and the targeting peptide-decorated **AD** in complex with a non-silencing siRNA (nssiRNA/**AD**/E_16_G_6_RGDK) at different **AD** concentrations (corresponding to nssiRNA concentrations of 10, 20, 50, 100, and 200 nM, respectively). In all these experiments, the siRNA/**AD**/E_16_G_6_RGDK nanoassemblies were prepared using 50 nM siRNA, siRNA/**AD** N/P = 10, and **AD**/E_16_G_6_RGDK molar ratio = 5. Adapted from [8] with the permission of the American Chemical Society, 2018.

Following siRNA delivery to PC-3 cells with the peptide-decorated **AD** nanomicelles, a drastic anticancer activity was observed again using only a 20 nM siRNA concentration (Figure 16b). This corresponds to an effect of 30% higher than that achieved with the pristine **AD** nanovectors. Concomitantly, the **AD**/E_16_G_6_RGDK nanoassemblies exhibited a safe toxicity profile, similar to that presented by the **AD** micelles (Figure A4), and in addition to its notable improvement in hemolytic effects (Figure A4c). All these data led the authors to perform the final testing of the siRNA/AD/E16G6RGDK nanoparticles in vivo.

### 4.3. In Vivo Targeted siRNA Delivery Performance of Double-Tail, Dual Targeting Self-Assembling Amphiphilic Dendron AD/E_16_G_6_RGDK

**Figure 17:**
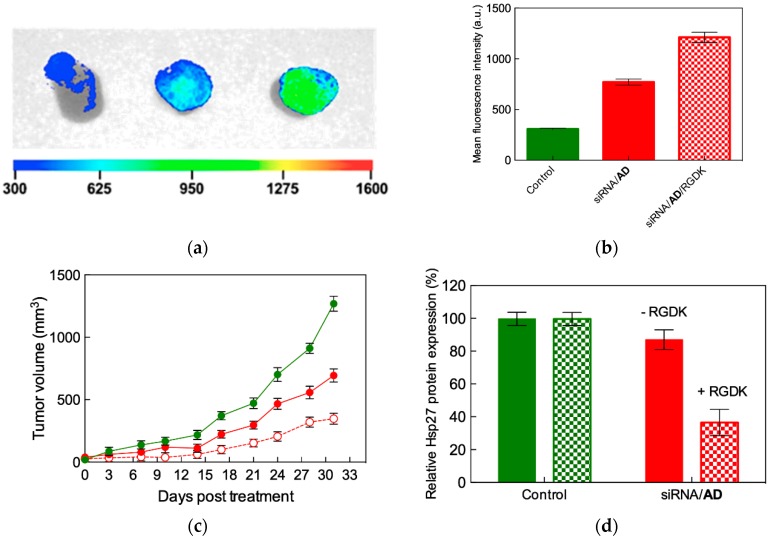
(**a**) Ex vivo fluorescence images obtained from tumor isolated from PC-3 xenograft mice treated with PBS buffer (delivery medium used as control, left), siRNA/**AD** (center), and siRNA/**AD**/E_16_G_6_RGDK nanoparticles (right). (**b**) Quantification of the mean fluorescence intensity for the images in panel **a**. PBS buffer (**c**) Tumor growth in untreated animals (green filled symbols), and in animals treated with siRNA/**AD** (red filled symbols). (**d**) In vivo knockdown of Hsp27 with siRNA/**AD** (-RGKD) and siRNA/**AD**/E_16_G_6_RGDK (+ RGDK) nanoparticles. Untreated animals were used as the control. The treatments (0.25 mg/kg siRNA, siRNA/**AD** N/P = 10, and **AD**/E_16_G_6_RGDK molar ratio = 5) were administered by intravenous injection twice per week. Adapted from [8] with permission of the American Chemical Society, 2018.

The in vivo gene silencing and anticancer effects of the peptide-decorated **AD** nanomicelles was investigated again using PC-3 xenograft nude mice. The first analysis was conducted to verify their enhanced tumor targeting ability with respect to the undecorated **AD** nanovectors using a fluorescent-labeled Hsp27 siRNA. Accordingly, ex vivo imaging of isolated tumors and the quantification of the relevant mean fluorescence intensity were performed (Figure 17a,b), from which the substantially higher tumor accumulation of the targeted nanoparticles was substantiated.

After 4 weeks of mice treatment with a very low siRNA concentration (0.25 mg/kg)—12-fold less than the conventional concentration used for siRNA delivery in mice—both the tumor volume and the corresponding levels of Hsp27 protein were drastically reduced when siRNA was administered via the peptide-decorate **AD** nanovectors, as shown in Figure 17c,d. In particular, at day 31 after treatment with the siRNA/**AD**/E_16_G_6_RGDK nanoparticles, tumor growth was reduced by 73% with respect to the control and by 50% relative to siRNA/**AD** treatment (Figure 17c). Consistently, Hsp27 expression in tumors was reduced by 65% and 41% upon siRNA treatment with the targeted nanovectors with respect to the control and **AD**-delivered siRNA, respectively (Figure 17d).

These highly promising results, coupled with the excellent in vivo toxicity profile exhibited by the **AD**/E_16_G_6_RGDK nanocarriers confirmed the high potential for these targeting nanosystems as safe and efficient siRNA delivery, gene silencing and consequent anticancer nanotherapeutics.

## 5. Conclusions

As defined by Nature.com [29], “self-assembly is the process by which an organized structure spontaneously forms from individual components, as a result of specific, local interactions among the components. When the constitutive components are molecules, the process is termed molecular self-assembly”. A key feature of molecular self-assembly is the multivalent, cooperative and synergistic nature of the intermolecular interactions leading to the organization of individual molecular entities into well-defined nanosized structures. This approach presents at least two major conceptual advantages. The first relies on the evidence that the driving forces governing self-assembly lead to the formation of the nano-objects virtually with no flaws, as interactions between the nanomicelle building blocks are mediated by specific molecular recognition ultimately resulting in complex and ordered nanoscale structures. The second, by no means of less important benefit to self-assembly is that very small amounts of material are required to accomplish the process.

In the companion paper [9], the authors presented the design, synthesis and gene silencing activity of modified PAMAM-based covalent nanovectors for siRNA delivery in cancer therapeutics. However, high generation dendrimer synthesis is extremely laborious and time-consuming, since the final product purification is difficult and hampered by the presence of highly similar side products. Thus, notwithstanding the highly promising results achieved with these molecules, the difficulties inherent in large-scale good manufacturing practice (GMP) production of high generation dendrimers led the authors to explore the potential of self-assembly in the design of new efficient siRNA nanocarriers.

Thus, as presented in this second short review, the small PAMAM-based amphiphilic dendron **4**, composed by a PAMAM head and a C_18_-long hydrocarbon tail, was initially developed. This was safe and effective in delivering siRNA both in vitro and in vivo. The natural evolution of **4** was its double-tail counterpart **AD**. The in vivo gene silencing effect obtained with siRNA delivered by **AD** nanomicelles in cancer treatment was comparable to that obtained with its precursor dendron **4**. However, **AD** nanovectors were also capable of eliciting gene silencing effects in the highly challenging and treatment-refectory human primary cells and stem cells.

The RNAi results of both the amphiphilic dendrons **4** and **AD** could be mainly attributed to passive targeting via the EPR effect in addition to their excellent siRNA delivery and endosomal escape capabilities. The authors focused on equipping **AD** with the dual E_16_G_6_RGDK targeting peptide, with the final goal of endowing the resulting nanovectors with cancer cell-directing specificity and, accordingly, higher anticancer activity. Compared to **AD**, the peptide-decorated **AD** nanomicelles exhibited more than 10-fold greater in vivo RNAi at significantly lower siRNA doses with respect to both covalent PAMAM dendrimers [9] and the non-targeted **AD** nanovectors. Our current activity in the field is progressing along this line, with the computer-assisted design of new targeting moieties for decorating self-assembling nanovectors in the development of gene silencing-based personalized medicine against different highly challenging and deadly (e.g., glioblastoma and pancreatic) human cancers.

## Figures and Tables

**Table 1:** Micelle aggregation number (N_agg_), surface charge density (σ_m_, *e*/nm^2^), diameter (D, nm), critical micelle concentration (CMC, μM) and free energy of micellization (ΔG_m_, kJ/mol) for dendrons **1**–**6** as predicted by computer simulations. The corresponding experimental values of the micelle diameter (D_exp_, nm) and critical micelle concentration (CMC_exp_, μM) are also reported. Adapted from [7] with the permission of John Wiley and Sons.

Dendron	N_agg_	D	D_exp_	CMC	CMC_exp_	ΔG_m_
**1**	5	5.6 ± 0.1	-	415	398 ± 22	−38.7
**2**	6	5.9 ± 0.1	-	127	116 ± 11	−44.4
**3**	7	6.2 ± 0.1	6.6 ± 0.1	37.2	49.9 ± 3.3	−50.5
**4**	8	6.4 ± 0.1	6.8 ± 0.1	11.4	15.6 ± 1.1	−56.4
**5**	10	7.1 ± 0.2	7.3 ± 0.1	9.13	11.2 ± 0.6	−57.5
**6**	13	8.1 ± 0.2	7.8 ± 0.2	8.50	8.24 ± 3.8	−57.8

**Table 2:** The free energy of micellization (ΔG_bind_, kcal/mol), number of effective charges (N_eff_), effective free energy of binding (ΔG_bind_,_eff_, kcal/mol), and effective-charge-normalized free energy of binding (ΔG_bind_,_eff_/N_eff_) between siRNA and dendrons **1**–**6** as derived from molecular simulations. The last column reports the siRNA/dendron binding data (C_50_, μM) obtained from ethidium bromide (EB) displacement assays. Adapted from [7] with the permission of John Wiley and Sons, 2016.

Dendron	ΔG_bind_	N_eff_	ΔG_bind,eff_	ΔG_bind,eff_/N_eff_	C_50_
**1**	−8.23 ± 0.91	13 ± 1	−2.47 ± 0.32	−0.19 ± 0.03	-
**2**	−13.4 ± 1.1	18 ± 1	−4.30 ± 0.45	−0.24 ± 0.03	42.0 ± 2.0
**3**	−17.1 ± 1.3	22 ± 1	−6.00 ± 0.36	−0.27 ± 0.02	16.9 ± 0.5
**4**	−22.6 ± 1.2	31 ± 1	−12.7 ± 0.41	−0.41 ± 0.04	7.0 ± 0.3
**5**	−46.6 ± 1.6	49 ± 1	−28.9 ± 0.70	−0.59 ± 0.04	5.3 ± 0.4
**6**	−57.8 ± 2.3	62 ± 1	−34.7 ± 0.62	−0.56 ± 0.05	4.8 ± 0.1

## Supplementary Materials

The following are available online at https://www.mdpi.com/1999-4923/11/7/324/s1, Figure S1. Schematic representation of the coarse-grained DPD model of the AD dendron. The different bead types are colored as follows: RC, dark magenta; R, plum; L, dark turquoise; G, chartreuse; C, light gray. Table S1. Example of DPD interaction parameters used to simulate the self-assembling of the amphiphilic dendron AD per se and in the presence of siRNA molecules.
