# Supplementary material for: Evolution from Covalent to Self-Assembled PAMAM-Based Dendrimers as Nanovectors for siRNA Delivery in Cancer by Coupled in Silico-Experimental Studies. Part II: Self-Assembled siRNA Nanocarriers
Source: Pharmaceutics. 2019 Jul 10;11(7):324. doi: 10.3390/pharmaceutics11070324 (PMC6680776; doi:10.3390/pharmaceutics11070324)
Supplement: Supplementary file 1 [file pharmaceutics-11-00324-s001.pdf]

pharmaceutics

MDPI

Supplementary Materials: Evolution from Covalent to Self-Assembled PAMAM-Based Dendrimers as Nanovectors for siRNA Delivery in Cancer by Coupled in Silico-Experimental Studies. Part II: Self-Assembled siRNA Nanocarriers

Erik Laurini, Domenico Marson, Suzana Aulic, Maurizio Fermeglia and Sabrina Pricl

Mesoscale Simulations of Amphiphilic Dendron Self-Assembling *per se* and in the Presence of siRNA Molecules

In the works described in this review, the mesoscopic Dissipative Particle Dynamics (DPD) simulation technique was employed. In DPD, a particle-based mesoscale technique [1], a number of particles are coarse-grained into fluid elements, called beads. These DPD beads interact via pairwise additive interactions that locally conserve momentum, a necessary condition for a correct description of hydrodynamics, while retaining essential information about the structural and physical-chemical properties of the system components. An advantageous feature of DPD is that it employs soft repulsive interactions between the beads, thereby allowing for larger integration time steps than in a typical molecular dynamics using for example Lennard-Jones interactions. Thus, time and length scales much larger (up to microseconds range) than those in atomistic molecular dynamics simulations can be accessed.

There are three types of forces between dissipative particles. The first type is a conservative force ($\boldsymbol{F}_{ij,C}$) deriving from a soft potential that tries to capture the effects of the "pressure" between different particles. The second type of force is a friction force ($\boldsymbol{F}_{ij,D}$) between the particles that wants to describe the viscous resistance in a real fluid. This force tries to reduce velocity differences between dissipative particles. Finally, there is a stochastic force ($\boldsymbol{F}_{ij,R}$) that describes the degrees of freedom that have been eliminated from the description in the coarse-graining process. This stochastic force will be responsible for the Brownian motion of (macro)molecules and colloidal particles simulated with DPD:

$$d\boldsymbol{r}_i(t) / dt = \boldsymbol{p}_i(t) / m_i \quad \text{(SI1)}$$

$$d\boldsymbol{p}_i(t) / dt = f_i(t) \quad \text{(SI2)}$$

where $\boldsymbol{r}_i(t)$, $\boldsymbol{p}_i(t)$, m_i, and $f_i(t)$ are the position, momentum, mass and net force of particle i, respectively. $f_i(t)$ is given as the sum of three different forces: the conservative force $\boldsymbol{F}_{ij,C}$, the dissipative force $\boldsymbol{F}_{ij,D}$, and the random force $\boldsymbol{F}_{ij,R}$:

$$f_i(t) = \Sigma_{i \neq j} \boldsymbol{F}_{ij,C} + \boldsymbol{F}_{ij,D} + \boldsymbol{F}_{ij,R} \quad \text{(SI3)}$$

All forces are pairwise and lay along the line joining two interacting particles i and j. The expressions for the forces are given by the following equations:

$$\boldsymbol{F}_{ij,C} = a_{ij} (1 - r_{ij} / r_c) \times \boldsymbol{r}_{ij} / r_{ij} \quad \text{(SI4)}$$

$$\boldsymbol{F}_{ij,D} = -\gamma_{ij}\ \omega^D(r_{ij}) [(\boldsymbol{r}_{ij} / r_{ij}) \times \boldsymbol{v}_{ij}] (\boldsymbol{r}_{ij} / r_{ij}) \quad \text{(SI5)}$$

$$\boldsymbol{F}_{ij,R} = \sigma_{ij}\ \omega^R(r_{ij}) (\quad _{ij} / \quad t^{0.5}) (\boldsymbol{r}_{ij} / r_{ij}) \quad \text{(SI6)}$$

where a_{ij} is the maximum repulsion parameter between particle i and j, $\boldsymbol{r}_{ij} = \boldsymbol{r}_i - \boldsymbol{r}_j$ is the vector joining beads i and j, $r_{ij} = |\boldsymbol{r}_{ij}|$ is the distance between particle i and j, $\boldsymbol{v}_{ij} = \boldsymbol{v}_i - \boldsymbol{v}_j$ is the relative velocity, and $\boldsymbol{v}_i = \boldsymbol{p}_i / m_i$. All the above forces acts within the cut-off radius r_c, which basically constitutes the length scale of the entire system. γ_{ij} is a friction coefficient, σ_{ij} the noise amplitude, ζ_{ij} a Gaussian random number with a zero mean and a unit variance chosen independently for each pair of particles, and Δt is the time step in the simulation. $\omega^D(r_{ij})$ and $\omega^R(r_{ij})$ are weight functions vanishing for distance greater than r_c.

In DPD, molecules are built by tying beads together using Hookean springs with the potential given by:

$$U_{bb}(i, i+1) = \tfrac{1}{2} k_{bb} (r_{i,i+1} - l_0)^2 \qquad \text{(SI7)}$$

where i, $i+1$ label adjacent beads in the molecule. The spring constant, k_{bb}, and unstretched length l_0, are chosen so as to fix the average bond length to a desired value. Chain stiffness is modeled by a three body potential acting between adjacent bead triples in a row using equation (SI8):

$$U_{bbb}(i-1,i,i+1) = k_{bbb} (1-\cos (\quad - \quad _0)) \qquad \text{(SI8)}$$

in which the angle   is defined by the scalar product of the two bonds connecting the pair of adjacent beads $i-1$, i, and $i+1$. An example of the DPD model is given in Figure S1for the amphiphilic dendron **AD** [2].

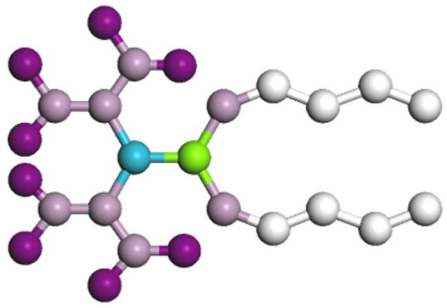

Figure S1. Schematic representation of the coarse-grained DPD model of the AD dendron. The different bead types are colored as follows: RC, dark magenta; R, plum; L, dark turquoise; G, chartreuse; C, light gray.

In the **AD** amphiphilic dendron case taken as example, a neutral bead types C was identified as the hydrophobic chain building block. A neutral bead type R and a positively charged bead type RC were employed for the non-protonated and terminal charged repeating unit of the **AD** dendron, respectively. Two further bead type, G and L, the first linking the hydrophilic and hydrophobic parts together, and the second representing the dendron focal point, were also employed.

Solvent molecules were simulated by single bead types W, and an appropriate number of counterions of a charge of ± 1 were added to preserve charge neutrality and to account for the experimental solution ionic strength. The inclusion of explicit counterions was necessary because counterion condensation and the interactions between the counterions and the charged groups may affect the complex structure to a certain extent.

Standard DPD particles were used to represent the siRNA chain beads, and the siRNA molecule was modeled as a self-avoiding string of 24 DPD S particles which are sequentially connected through a wormlike chain spring. Moreover, in order to correctly derive the electrostatic interactions between charged beads, the electrostatic force between two charged beads i and j was analyzed following solving the electrostatic field by smearing the charges over a lattice grid, the size of which is determined by a balance between the fast implementation and the correct representation of the electrostatic field.

Having set the reference volume of one bead and the density of the system to a value of ρ=3, the appropriate number of **AD** and siRNA molecules were added to the simulation box in order to fit

experimental concentrations and to reproduce an N/P ratio of 10. 7×10^6 time steps were performed in each DPD run, with a total physical time of each calculation being approximately 3s, to obtain the self-assembled morphology of the dendron alone and in presence of siRNA.

The intra- and intermolecular interactions between DPD particles are expressed by the conservative parameter a_{ij}, defined by equation (SI4). This quantity accounts for the underlying chemistry of the system considered. In this work, we employed a well-validated strategy that correlates the interaction energies estimated from atomistic molecular dynamics (MD) simulations to the mesoscale a_{ij} parameter values [3]. Following this computational recipe, the interaction energies between the solvated 1:1 **AD**/siRNA systems estimated using atomistic molecular dynamics simulations [3] were rescaled onto the corresponding mesoscale segments adapting the procedure described in detail in [4]. The bead-bead interaction parameter for water was set equal to $a_{ww} = 25$ in agreement with the correct value of DPD density $\rho = 3$. The maximum level of hydrophobic/hydrophilic repulsion was captured by setting the interaction parameter a_{ij} between the water bead W and the hydrocarbon tail bead C as 82. The counterions were set to have the interaction parameters of water. Once these parameters were assigned, all the remaining bead-bead interaction parameters for the DPD simulations were easily obtained, starting from the atomistic interaction energies values, as shown in Table S1.

Table 1. Example of DPD interaction parameters used to simulate the self-assembling of the amphiphilic dendron AD per se and in the presence of siRNA molecules.

	**RC**	**R**	**L**	**G**	**C**	**S**	**W**
RC	41						
R	31	27					
L	33	34	31				
G	46	42	47	26			
C	80	82	40	60	22		
S	10	35	38	33	74	45	
W	22	31	65	29	82	20	25

In Silico Free Energy of Micellization and Critical Micellar Concentration

From an energetic standpoint, the change in Gibbs free energy of transfer of a single amphiphilic molecule from the monomeric state to a micelle of aggregation number N_{agg}, commonly called the free energy of micellization ΔG_{mic}, can be modeled as consisting of a hydrophobic part, $\Delta G_{mic,h}$, and an electrostatic part, $\Delta G_{mic,e}$, so that $\Delta G_{mic} = \Delta G_{mic,h} + \Delta G_{mic,e}$. The hydrophobic part stems primarily from the favorable energy of transfer of the hydrocarbon moieties from the aqueous phase to the micellar phase, and, secondarily, from the unfavorable residual interfacial contact of water with the apolar components within the micelles. The electrostatic part of ΔG_{mic} arises from the repulsion between the ionic head groups within the micellar shell. In the work discussed in this review [5], we calculated the values of ΔG_{mic} for the amphiphilic dendrons following the theory described in [6].

References

1. Groot, R.D.; Warren, P.B. J. Dissipative particle dynamics: Bridging the gap between atomistic and mesoscopic simulation. *J. Chem. Phys.* **1997**, *107*, 4423–4443.
2. Liu, X.; Zhou, J.; Yu, T.; Chen, C.; Cheng, Q.; Sengupt, K.; Huang, Y.; Li, H.; Liu, C.; Wang, Y.; et al. Adaptive amphiphilic dendrimer-based nanoassemblies as robust and versatile siRNA delivery systems. *Angew. Chem. Int. Ed. Engl.* **2014**, *53*, 11822–11827.
3. Posocco, P.; Laurini, E.; Dal Col, V.; Marson, D.; Karatasos, K.; Fermeglia, M., Pricl, S. Tell me something that I do not know. Multiscale molecular modeling of dendrimer/dendron organization and self-assembly in gene therapy. *Curr. Med. Chem.* **2012**, *19*, 5062–5087.
4. Scocchi, G.; Posocco, P.; Handgraaf, J.-W.; Fraaije, J. G.E.M.; Fermeglia, M.; Pricl, S.; A complete multiscale modeling approach for polymer–clay nanocomposites. *Chem. Eur. J.* **2009**, *15*, 7586–7592.

5. Chen, C.; Posocco, P.; Liu, X.; Cheng, Q.; Laurini, E.; Zhou, J.; Liu, C.; Wang, Y.; Tang, J.; Dal Col, V.; et al. Mastering dendrimer self-assembly for efficient siRNA delivery: From conceptual design to in vivo efficient gene silencing. *Small* **2016**, *12*, 3667–3676.
6. Patrickios, C.S. Micellization model for multivalent ionic surfactants. *J. Phys. Chem.* **1995**, *99*, 17437–17441.
